# In the right place, at the right time: the integration of bacteria into the Plankton Ecology Group model

**DOI:** 10.1186/s40168-023-01522-0

**Published:** 2023-05-20

**Authors:** Hongjae Park, Tanja Shabarova, Michaela M. Salcher, Lenka Kosová, Pavel Rychtecký, Indranil Mukherjee, Karel Šimek, Petr Porcal, Jaromír Seďa, Petr Znachor, Vojtěch Kasalický

**Affiliations:** 1 Institute of Hydrobiology, Biology Centre of the Czech Academy of Sciences, České Budějovice, Czech Republic; 2 Faculty of Science, University of South Bohemia, České Budějovice, Czech Republic

**Keywords:** Freshwater reservoirs, Microbial communities, Spatiotemporal dynamics, Microdiversity, PEG model

## Abstract

**Background:**

Planktonic microbial communities have critical impacts on the pelagic food web and water quality status in freshwater ecosystems, yet no general model of bacterial community assembly linked to higher trophic levels and hydrodynamics has been assessed. In this study, we utilized a 2-year survey of planktonic communities from bacteria to zooplankton in three freshwater reservoirs to investigate their spatiotemporal dynamics.

**Results:**

We observed site-specific occurrence and microdiversification of bacteria in lacustrine and riverine environments, as well as in deep hypolimnia. Moreover, we determined recurrent bacterial seasonal patterns driven by both biotic and abiotic conditions, which could be integrated into the well-known Plankton Ecology Group (PEG) model describing primarily the seasonalities of larger plankton groups. Importantly, bacteria with different ecological potentials showed finely coordinated successions affiliated with four seasonal phases, including the spring bloom dominated by fast-growing opportunists, the clear-water phase associated with oligotrophic ultramicrobacteria, the summer phase characterized by phytoplankton bloom-associated bacteria, and the fall/winter phase driven by decay-specialists.

**Conclusions:**

Our findings elucidate the major principles driving the spatiotemporal microbial community distribution in freshwater ecosystems. We suggest an extension to the original PEG model by integrating new findings on recurrent bacterial seasonal trends.

Video Abstract

**Supplementary Information:**

The online version contains supplementary material available at 10.1186/s40168-023-01522-0.

## Background

Freshwater reservoirs are man-made waterbodies created by damming of rivers. Dams interrupt the natural continuity of water flows, thereby holding water for multiple purposes, including energy generation, drinking water supply, or flood protection [1]. Since breaking the natural flow regime can significantly alter influential hydrodynamics such as water-level fluctuation and water retention time, the ecological consequences of building a reservoir can be manifold [2]. The most pronounced effect of building a dam is the intensification of longitudinal gradients which in natural rivers tend to be developed gradually across hundreds or even thousands of kilometers [3]. The reservoir zonation model proposed by Thornton et al. [4] described the artificial freshwater environment into three distinct zones: riverine (inflow), transition, and lacustrine (near the dam). According to the model, water velocity and nutrient concentration decrease from inflow to the dam, with a consequent increase in water residence time and transparency. In lacustrine parts of deep temperate reservoirs, the water column is often thermally stratified during vegetation seasons with remarkable gradients of temperature, oxygen, or nutrients [5]. This leads to the development of warm epilimnion, metalimnion (thermocline), and cold hypolimnion, which act as barriers to the exchange of heat, oxygen, and nutrients [6]. Such environmental differences along both longitudinal and vertical axes provide unique environments hosting locally-specific microbial inhabitants [7–10].

Planktonic community dynamics in freshwater reservoirs have been thoroughly described mainly at the level of phytoplankton [11, 12] or zooplankton [13]. The maximum phytoplankton biomass is expected in a transition zone, while limited light availability (due to high turbidity) in riverine and nutrient depletion in lacustrine zones hamper phytoplankton development [14]. The lack of bacterivorous microbial eukaryotes in the upstream reservoir parts clearly distinguishes turbulent riverine environments from stagnant water in lacustrine regions [15, 16]. The well-known Plankton Ecology Group (PEG) model [17], which describes the seasonal succession patterns of phyto- (photo- and mixotrophic algae and cyanobacteria) and zooplankton (mainly heterotrophic nanoflagellates (HNF), ciliates, rotifers, copepods, and cladocerans) communities in lakes, could be broadly adopted in other aquatic ecosystems including the lacustrine parts of freshwater reservoirs (representing a lake-like ecosystem) and marine pelagic environments [13, 18]. However, unlike phyto- and zooplankton, no general model for the seasonality of heterotrophic bacteria in freshwater ecosystems has so far been developed, given their inherent microdiversity and rapid generation times [19], as well as the challenges in conducting long-term studies including multiple trophic levels [20].

The meta-analyses of all reported 16S rRNA sequences in 2002 [21] and 2011 [22] recovered 21 bacterial phyla from diverse freshwater environments. Freshwater-specific bacterial taxa were represented by the five most common phyla, including Proteobacteria, Actinobacteria, Bacteroidota, Cyanobacteria, and Verrucomicrobia which contribute up to 50% of the total sequences [22]. Our knowledge of ecological niche partitioning of freshwater bacteria in reservoirs is still fragmentary since most previous studies provided only snapshots of community compositions in a narrow time frame [8, 23, 24] and limited space [25, 26]. A few long-term and continuous sampling campaigns at the annual scale focused on specific taxonomic populations [27–30], leaving many blind spots.

This study presents a microbial survey of three canyon-shaped reservoirs with a 3-week sampling frequency. We investigated planktonic community dynamics (including bacteria, phytoplankton, and zooplankton) and physicochemical parameters over a wide trophic gradient (oligotrophic to meso-eutrophic), different strata (epilimnion and hypolimnion), and longitudinal planes (Inflow, Middle, and Dam stations) for two consecutive years. We identified (i) bacterial populations specific for particular longitudinal zones and vertical layers, (ii) their microdiversification connected to spatial localization, and (iii) recurrent bacterial seasonal patterns, which we used to extend the original PEG model.

## Methods

### Defining terms in planktonic food web

The classic characterization of plankton communities into phytoplankton and zooplankton can be considered inadequate since mixotrophic protists can contribute to both phyto- and zooplankton communities [31]. Cell-size dependent grouping of zooplankton into micro- (20–200 µm) and mesozooplankton (0.2–20 mm) is also problematic since HNF (2–20 µm) often do not fall into either category [32], and ciliates (10 µm–4 mm) have a variable size range [33]. However, to allow better integration of our results into the original PEG model, we still adopted the classic terminology. In this study, we use the term phytoplankton for pigmented organisms comprising all algal groups, including photo- and mixotrophic protists and cyanobacteria. We use the term zooplankton for those having grazing potentials in the planktonic food web. The original PEG model mainly referred to HNF, ciliates, rotifers, copepods, and cladocerans as important zooplankton representatives [17]. In our work, we followed the dynamics of all these groups except rotifers as the applied methods in this study did not allow us to evaluate the densities of rotifers accurately.

### Study sites and sample collection for DNA isolation

**Fig. 1:**
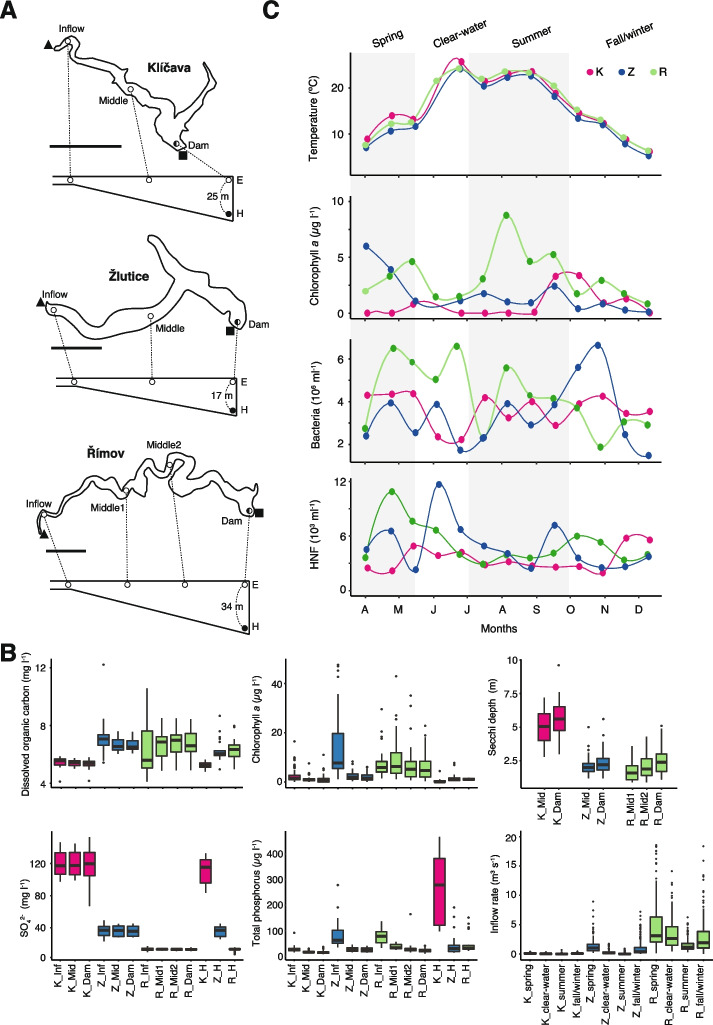
A 2-year microbial survey across three canyon-shaped reservoirs. **A** Maps of Klíčava (top), Žlutice (middle), and Římov (bottom) reservoirs. Sampling stations are indicated as open (epilimnion) and closed (hypolimnion) circles. The location of water inflows and dams are indicated by closed triangles and squares, respectively, outside the maps. The bold lines represent a scale of 1 km. **B** Comparison of the key environmental profiles. Each box plot includes all the samples collected during the study. The lower and upper edges of the boxplots correspond to the first and third quartiles, the whiskers extend to the largest or smallest value at 1.5 times the interquartile, and the black bars across the box represent median values. Dots that exist beyond one of the whiskers represent outliers. **C** Identification of four successional seasonal phases (spring, clear-water, summer, and fall/winter) at the lacustrine regions based on key environmental profiles from the year 2019 (data from the year 2018 is not included). K: Klíčava, Z: Žlutice, R: Římov, E: Epilimnion, H: Hypolimnion

Three reservoirs with different trophic statuses (oligotrophic Klíčava, and meso-eutrophic Žlutice and Římov) located in the Czech Republic were selected for the 2-year microbial survey (Table 1; Fig. 1A). Samples were collected at the surface (0.5 m depth) from the Inflow, Middle (two Middle for the Římov reservoir considering its prolonged morphology), and Dam stations. Hypolimnion samples were collected at the Dam area from the 25, 17, and 34 m depths for Klíčava, Žlutice, and Římov, respectively. The sampling campaign (*n* = 310) was conducted at a 3-week interval from May 2018 to December 2019, except for the periods when the reservoirs were covered by ice (January–February). A Friedinger-type sampler (Šramhauser s.r.o., Dolní Bukovsko, CZ) was used to collect two liters of water at each sampling point. All samples were prefiltered through a 40-µm nylon net to remove coarse particles and bigger organisms. Subsequently, vacuum filtration was used to collect biomass on 0.22 μm polyethersulfone membrane filters (Millipore, Merck, Darmstadt, DE). The filters (containing both free-living and particle-attached bacteria) were stored at − 80 °C until further processing.

**Table 1:** Locations and the hydrological conditions of three targeted dam reservoirs during the studied period

	Klíčava	Žlutice	Římov
GPS coordinates (dam site)	50.0649169N, 13.9337606E	50.0879269N, 13.1279789E	48.8475817N, 14.4902242E
Trophic status	Oligotrophic	Meso-eutrophic	Meso-eutrophic
Total surface area (km^2^)	0.57	1.20	1.89
Mean depth (m)	10.9	7.6	15
Maximum depth (m)	30.3	20.2	41.4
Water retention time (year)	3.1	0.43	0.31
Mean inflow (m^3^/s)	0.06	0.68	2.93

### Environmental measures

Vertical profiles of temperature, pH, conductivity, and dissolved oxygen (DO) concentration were measured at the sampling sites by a YSI EXO II multiparameter probe (YSI, Yellow Springs, OH, USA). A multi-wavelength submersible fluorescence probe (FluoroProbe, bbe-Moldaence, Kiel, DE) was employed to measure chlorophyll *a* concentration. The measured data could be converted into the amount of chlorophyll *a* per liter of water using the original software provided with the probe. Daily data on inflow rates were obtained from the Vltava River Authority.

For additional physicochemical parameters, water samples (100 ml) were delivered to the laboratory in a thermobox. Chlorophyll *a* concentration was determined spectrophotometrically after extraction with ethanol (Sigma-Aldrich, St. Louis, MO, USA) [34]. Concentrations of cations (Na^+^, K^+^, Ca^2+^, and NH_4_^+^) and anions (Cl^–^, SO_4_^2–^, F^–^, NO_2_^–^, and NO_3_^–^) were determined by Dionex IC25 ion chromatography (Dionex, Sunnyvale, CA, USA) using aliquots of water filtered onto 0.45 µm glass fiber filters (Fisher Scientific, Tustin, CA, USA). Dissolved reactive phosphorus (DRP) was determined by the molybdate method [35]. Total and dissolved phosphorus (TP and DP, respectively) were measured using a modified protocol of [36] with sample preconcentration and perchloric acid digestion. Dissolved organic carbon (DOC) concentration was measured as a non-purgeable organic carbon by catalytic combustion at 680 °C (Elementar, Hanau, DE). Samples were acidified with 1M HCl (Sigma-Aldrich, St. Louis, MO, USA) to pH < 4 and air purged for 3 min before the analysis. Dissolved nitrogen (DN) concentrations were obtained using a vario TOC cube (Elementar, Hanau, DE).

### Enumeration of planktonic organisms

The enumeration of planktonic organisms was carried out as previously described [37]. For bacterial enumeration, aliquots of water samples without prefiltration were fixed with formaldehyde (2 % final concentration). HNF and ciliate samples were fixed with Lugol-formaldehyde-thiosulfate method [38]. Bacteria and protists were both stained with DAPI (Thermo Fisher, Waltham, MA, USA) and counted on 0.22 μm and 1.0 μm polycarbonate membrane filters (Sterlitech, Kent, WA, USA), respectively, using epifluorescence microscopy [39]. Phytoplankton samples were preserved with acid Lugol solution. Species were identified and enumerated with the Utermöhl method [40] using an Olympus BX50 microscope (Olympus, Tokyo, JP). Using this method, we were able to count algal cells larger than 2 µm. The mean algal cell dimensions for biovolume calculation were obtained using the approximation of cell morphology to regular geometric shapes [41]. Crustaceans were collected by vertical hauls using an Apstein plankton net (200 μm) from the entire water columns. Samples were preserved in 4 % formaldehyde (Sigma-Aldrich, St Louis, MO, USA), and species abundances were determined microscopically [42].

### Genomic DNA extraction and 16S rRNA amplicon sequencing

Genomic DNA was extracted from the 0.22 µm polyethersulfone filters using a modified phenol:chloroform:isoamyl alcohol extraction method [43]. In brief, filters were thawed on ice and incubated with 0.7 ml of lysis buffer (50 mM Tris pH 8.0, 40 mM EDTA, and 1% SDS). Cells were lysed by adding 20 µl of lysozyme (40 mg ml^−1^; Serva, Heidelberg, DE) and Proteinase K (10 mg ml^−1^; Macherey-Nagel, Düren, DE). The samples were incubated at 55 °C for 30 min, followed by 37 °C for 1 h. An equal volume of phenol:chloroform:isoamyl alcohol (Sigma-Aldrich, St. Louis, MO, USA) was added to stop the cell lysis, and the samples were vortexed vigorously for 1 min. After centrifugation at 10,000×*g* for 10 min, the top aqueous layers were transferred to new 1.5 ml tubes. An equal volume of phenol:chloroform:isoamyl alcohol (Sigma-Aldrich, St. Louis, MO, USA) was added, and the samples were centrifuged again at 10,000×*g* for 10 min. The top aqueous phases were subsequently transferred to clean tubes, and the DNA was precipitated by adding 0.1 volume of 3 M sodium acetate (Sigma-Aldrich, St. Louis, MO, USA) and 0.6 volume of isopropanol (Sigma-Aldrich, St Louis, MO, USA). After centrifugation at 10,000×*g* for 20 min, the DNA pellets were washed with 500 µl of 70% ethanol (Sigma-Aldrich, St. Louis, MO, US), centrifuged again, and dissolved in 50 µl of TE. The amount of DNA was determined using a Qubit fluorometer (Invitrogen, Carlsbad, CA, USA). The primer pair 515F and 926R was used to generate paired-end amplicons covering V4-5 regions of the 16S rRNA sequence [44, 45]. Amplicons were sequenced using 2 × 250 chemistry under the Illumina Miseq platform (Illumina, San Diego, CA, USA). Library preparation and sequencing were performed at the Genome Research Core (GRC) at the University of Illinois at Chicago. A DNA extraction control and a commercial mock community (ZymoBIOMICS Microbial Community DNA Standard; Zymo Research, Irvine, CA, USA) were also sequenced in the same run.

### Data processing and analysis

The reads obtained from 16S rRNA amplicon sequencing were processed using the R package DADA2 v1.16.0 [46], following the standard pipeline available at https://benjjneb.github.io/dada2/tutorial.html. All merged sequence variants in the range of 400–420 bp in length were submitted to unsupervised oligotyping using Minimum Entropy Decomposition (MED) with minimum substantive abundance (-M) of 100 and maximum variation allowed (-V) of 4 [47]. Taxonomic classification of the sequences was performed using the TaxAss pipeline, which uses the freshwater-specific FreshTrain database [48] to first classify the sequences at the 98% identity threshold, and then the remaining sequences using the SILVA database v138 [49, 50]. Further analysis of the data was conducted using the R package phyloseq v1.28.0 [51]. Oligotypes with taxonomic assignments associated with eukaryotes, chloroplasts, and mitochondria were removed before rarefying the data to a common read depth of 8000 for each sample. Rarefaction diversity curves for individual samples were generated using ‘ggrare’ function in the R package ranacapa v0.1.0 [52]. Species accumulation curves were produced using the function ‘specaccum’ in the R package vegan v2.5-7 [53]. Hierarchical clustering of the samples using the Bray-Curtis dissimilarity analysis was conducted using the function ‘hclust’ (‘complete’ method) implemented in base R [54]. Before applying the clustering analysis, samples (*n* = 310) were clustered into 52 groups according to their collection sites (13 sampling stations) and seasons (four successional phases), and the average read counts for individual oligotypes were calculated. Indicator species analysis was performed using the R package indispecies v1.7.9 [55]. Maximum likelihood trees for the oligotypes affiliated with betIV-A and *Rhodoferax* were constructed using RAxML (randomized axelerated maximum likelihood with the general time-reversible substitution and gamma rate heterogeneity model GTR-GAMMA) [56] after alignment with MAFFT (Multiple Alignment using Fast Fourier Transform) v7 [57]. In order to integrate heterotrophic bacteria into the PEG model, bacterial seasonality was revealed by soft clustering using the R package Mfuzz v2.44.0 [58]. Bacterial groups whose maximum abundance was higher than 1% were used for this analysis and the number of clusters was set to four (one cluster for each successional seasonal phase). Plots and heatmaps were generated using the R package ggplot2 v3.3.5 [59] and pheatmap v1.0.12 (https://github.com/raivokolde/pheatmap). Association network analysis was performed using the R scripts available at https://github.com/RichieJu520/Co-occurrence_Network_Analysis [60]. Spearman’s correlation coefficient was calculated to identify strong correlations. Read abundance patterns from the dam epilimnion samples of all three reservoirs were used for the analysis. Network visualization and modular analysis was conducted using Gephi v0.9.2 [61].

## Results

### Environmental characteristics

To identify the key environmental factors in microbial community assembly, a large suite of physicochemical data was obtained over the study period (Figure S1–S4). Key environmental parameters from all three reservoirs are summarized in Fig. 1B. The surface water characteristics of the oligotrophic Klíčava differed substantially from the other two meso-eutrophic reservoirs. Klíčava showed the lowest DOC (5.32 ± 0.33 mg l^−1^, average ± s.d.) and chlorophyll *a* (0.84 ± 1.77 µg l^−1^) concentrations along with the highest water transparency (5.68 ± 1.49 m) and sulfate concentration (119.25 ± 20.71 mg l^−1^). Water retention time at the lacustrine part was the longest (2.3 years) in Klíčava, and much shorter (< 0.5 year; Table 1) in both Římov and Žlutice. Clear thermal stratification and water mixing were observed in all three reservoirs (Figure S3), while an anoxic bottom layer persisted in Klíčava throughout the whole year (Figure S4). This anoxia was associated with the remarkable accumulation of phosphorus (up to 434.7 µg l^−1^ of TP) at the hypolimnion of Klíčava (Fig. 1B). In Římov, the inflow rate was substantial (3.02 ± 3.22 m^3^ s^−1^), while in the other two reservoirs, the water discharges were relatively low (0.69 ± 1.09 m^3^ s^−1^ in Žlutice) or negligible (0.07 ± 0.07 m^3^ s^−1^ in Klíčava) (Fig. 1B).

According to the seasonal changes of the key environmental parameters, including water temperature and chlorophyll *a* concentration, we divided each year into four successional phases: spring bloom period (March–April), clear-water phase (May–June), summer (July–September), and fall/winter (October–February) (Fig. 1C). The spring bloom was characterized by the first peak of chlorophyll *a.* In accordance with the PEG model [17], phytoplankton peaks in the oligotrophic Klíčava were less prominent than those in other meso-eutrophic reservoirs (Fig. 1C). The delay of summer phytoplankton peak in Klíčava (mid September–October) was also notable. The seasonal patterns of total bacterial abundances and HNF in different reservoirs did not show strong correlations to each other; however, the spring maxima of bacteria in all reservoirs corresponded to the highest abundances of bacterivorous HNF. A marked decline of chlorophyll *a* concentration after the spring bloom resulted in the clear-water phase. The second peak of phytoplankton developed in summer during the strongest stratification of the water column (Figre S3). At the end of the year (fall/winter stage), a drop in temperature, chlorophyll *a*, and bacterial biomass was a common trend in all three reservoirs.

### Overview of bacterial community compositions

Using MED analysis, 10,851 oligotypes belonging to 566 different taxonomic groups were identified in 310 samples. Among them, 2112 (accounting for 43.6% of total reads) and 8736 (56.4% of total reads) oligotypes were classified using the freshwater-specific [62] and SILVA database [49, 50], respectively. Rarefaction diversity curves for each sample reached a plateau, indicating that the sequencing depth was sufficient to incorporate most of the bacterial populations present in a sample (Figure S5A). In addition, the species accumulation curves for the whole data set showed that the number of samples was large enough to cover the temporal variations in bacterial communities (Figure S5B).

**Fig. 2:**
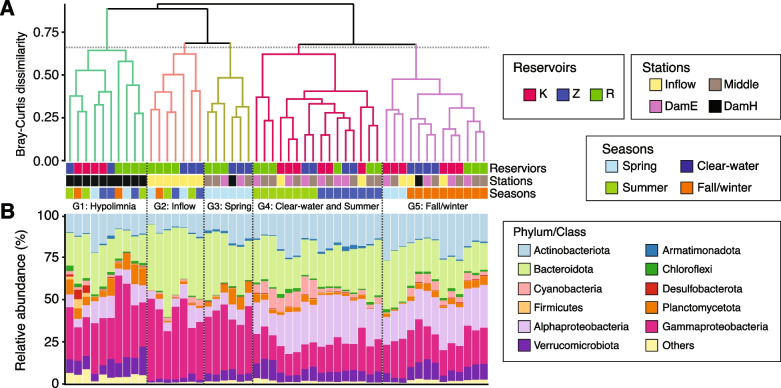
Hierarchical clustering and community compositions of the samples.** A** The dendrogram shows the clustering of the samples from three reservoirs (K: Klíčava, Z: Žlutice, and R: Římov) covering two water depths (E: Epilimnion and H:Hypolimnion), 13 sampling stations, and four successional seasonal phases (spring, clear-water, summer, and fall/winter) over 2 years. Samples (read counts averaged by collection sites and seasons) were assigned to each group (G1 to G5) by a cut-off value of 0.65. A hierarchical clustering of individual samples (*n* = 310) is also available in Figure S6. Below the dendrogram (**B**) shows the average relative proportions of the top 10 bacterial phyla

Hierarchical clustering of the samples (Fig. 2A) showed that the samples could be largely divided into five different groups (G1–G5). G1 was highly specific to the hypolimnion samples. This group could be further divided into Římov- and Klíčava-specific subgroups. Planctomycetota were more abundant in the hypolimnion of Římov, while Desulfubacterota and Firmicutes were specifically present in the hypolimnion of Klíčava (Fig. 2B). In both Římov and Žlutice, G2 and G3 were associated with the Inflow and spring samples, respectively. The compositions of these two groups were similar at the phylum level and characterized by the dominance of Bacteroidota and Gammaproteobacteria, and a low number of Alphaproteobacteria. G4 was composed of samples from the clear-water phase and summer. Higher proportions (up to 12%) of cyanobacteria were notable for the summer samples. G5 was mainly associated with fall/winter samples. At the phylum level, we observed little variation between the summer and fall/winter samples, implying that the community difference probably occurred at lower taxonomic levels.

### Spatial distribution and microdiversification of planktonic bacteria

**Fig. 3:**
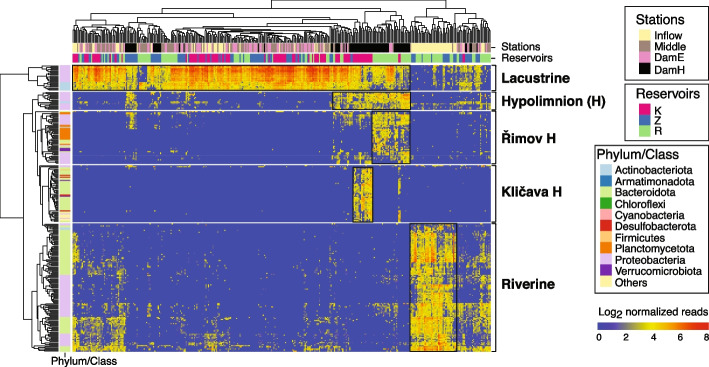
Spatial distribution of freshwater bacteria along different sampling stations and water depths. The heatmap shows the abundance patterns (log_2_ read count) of the indicator oligotypes (indicator value > 0.5, *p* value < 0.01). Columns and rows represent different samples and oligotypes, respectively. K: Klíčava, Z: Žlutice, R: Římov, E: Epilimnion, H: Hypolimnion

We also investigated the distribution of freshwater bacteria across different reservoir environments. Indicator species analysis was performed at the oligotype level (Table S1), and the relative proportions of the selected indicator strains (indicator value > 0.5 and *p* value < 0.01) are shown in a heatmap (Fig. 3). Five distinctive clusters for the lacustrine, riverine, hypolimnion, Klíčava-hypolimnion, and Římov-hypolimnion clearly demonstrated the spatial localization of freshwater bacteria. The lacustrine cluster (epilimnion at the dam) was composed of genome-streamlined ultramicrobacteria including LD12 (‘*Ca*. Fonsibacter’) [63], LD28 (‘*Ca*. Methylopumilus’) [29], and acI-B1 (‘*Ca*. Nanopelagicus’) [30]. A large riverine-specific cluster was composed of oligotypes classified as Flavo-A3, PnecC [64], Lhab-A3 [65], bacV, bacIII-A, and betI-A from Gammaproteobacteria and Bacteroidota. As common hypolimnion-specialists, oligotypes classified as Rhodo (*Rhodoferax*), acI-A7 (‘*Ca*. Planktophila vernalis’), *Nitrosospira*, *Methylobacter*, and unclassified Methylophilaceae were found. Oligotypes from unclassified Methylophilaceae were affiliated with betIV-A but could be distinguished from LD28 at the tribe level (98% identity cut-off). Klíčava showed the most distinctive community assembly in the hypolimnion. The presence of specific oligotypes affiliated with *Sulfuritalea*, *Sulfurimonas*, *Sulfuricurvum*, *Desulfatirhabdium*, and unclassified Desulfocapsaceae reflected a potential sulfur-oxidizing environment. A putative phosphorus-accumulating ‘*Ca*. Accumulibacter’ [66] was clearly associated with high phosphorus concentrations (Fig. 1B). We also noticed that a total of 16 novel oligotypes affiliated with Bacteroidetes_VC2.1_Bac22 group were present only in this environment. The cluster for Římov-hypolimnion was composed mainly of CL500-3 [67], unclassified Isosphaeraceae, and Gemmataceae from Planctomycetota, as well as *Methylobacter*, ‘*Ca*. Nitrotoga’, and unclassified Verrucomicrobiae.

**Fig. 4:**
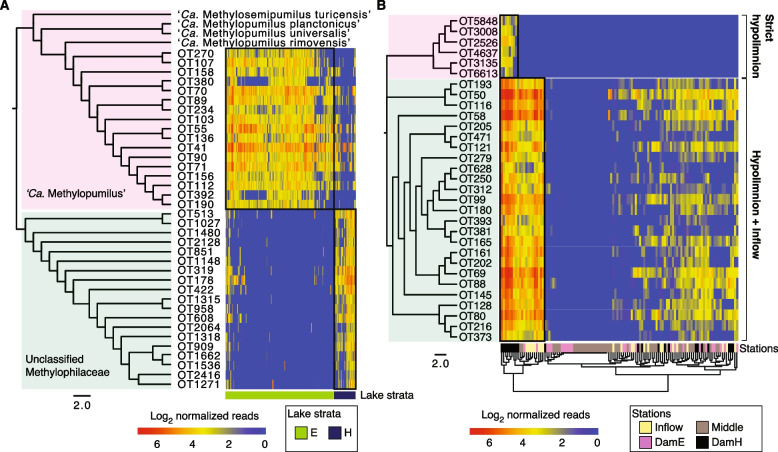
Microdiversification within betIV-A (**A**) and *Rhodoferax* (**B**). Maximum likelihood trees (on the left of each panel) of the 16S amplicon sequences divide the population into two groups. The scale bars at the bottom correspond to nucleotide substitutions per site. The heatmap (on the right) shows the abundance patterns (log_2_ read count) of the oligotypes. For the heatmap of *Rhodoferax* (B), samples only from the Římov reservoir were analyzed, since only Římov developed a clear riverine environment. Rows represent oligotypes and columns represent different samples color-coded by sampling depths (**A**) or stations (**B**). E**: **Epilimnion, H: Hypolimnion, OT: Oligotype

We found on average 19 oligotypes for individual tribes, giving the potential to reveal the existence of microdiversification related to ecological niche separation. Two bacterial groups gave positive signals on both criteria, phylogenetics and read abundance pattern. We first focused on 40 oligotypes affiliated with betIV-A. Phylogenetic analysis suggested a clear separation of the population into two groups (Fig. 4A). Oligotypes affiliated with LD28 showed a high similarity to previously described three ‘*Ca*. Methylopumilus’ species [68], while those affiliated with unclassified Methylophilaceae were phylogenetically distinct from others, thereby representing a novel phylogenetic group. The read abundance patterns clearly demonstrated the depth-dependent separation of these two groups. Another example of microdiversification was observed among 31 oligotypes affiliated with Rhodo (*Rhodoferax*) (Fig. 4B). The first group composed of 25 oligotypes was successfully colonizing both hypolimnion and riverine environments. The second group, however, was composed of six oligotypes that were strict hypolimnion specialists.

### Seasonal bacterial dynamics in the context of the PEG model

**Fig. 5:**
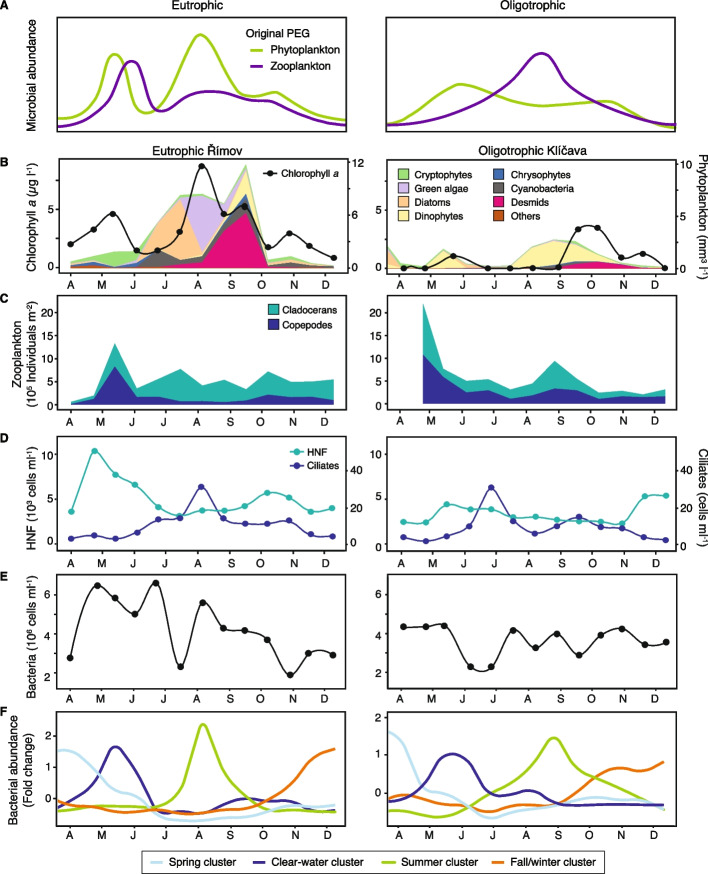
Annual succession patterns of planktonic organisms in reservoir ecosystems. **A** The original PEG model in eutrophic and oligotrophic conditions, **B** chlorophyll *a* concentration and phytoplankton biomass, **C** seasonal patterns of copepods and cladocerans, **D** seasonal patterns of HNF and ciliates, **E** total bacterial cell counts, **F** the average abundance changes of four seasonal clusters based on soft clustering analysis. Data from the year 2019 is presented (data from the previous year is shown additionally in Figure S7)

To integrate bacterial seasonality into the original PEG model (Fig. 5A), Římov and Klíčava were selected as the models of eutrophic and oligotrophic systems, respectively. Since Římov and Žlutice were both eutrophic, the shallowest reservoir Žlutice was not included for further analysis. In Římov and Klíčava, we additionally followed the seasonal succession of major phytoplankton (cryptophytes, green algae, diatoms, dinophytes, chrysophytes, desmids, and cyanobacteria) and zooplankton (HNF, ciliates, copepods, and cladocerans) groups. The seasonal succession of both phyto- and zooplankton generally followed the patterns outlined in the PEG model (Fig. 5B-D). In Římov, the maxima of copepods/cladocerans corresponded to the spring peak of phytoplankton. The phytoplankton community in this eutrophic environment was predominated by cryptophytes during the spring bloom, and diatoms, green algae, and desmids at the second summer peak. In Klíčava, a pronounced copepods/cladocerans peak during the spring bloom was notable. The most abundant phytoplankton group in this oligotrophic reservoir was dinophytes. In both reservoirs, ciliate peaks developed in summer, which appeared to have a strong influence on the bacterial biomass (Fig. 5D-E).

To get access to bacterial seasonality, oligotypes were clustered into the lowest taxonomic assignments (tribe-level or higher), and the resulting 58 bacterial groups with maximum relative proportions higher than 1% were examined. We used an unsupervised approach based on soft clustering to assign them into four seasonal clusters (Table S2, Fig. 5F). In both Římov and Klíčava, spring clusters were mainly composed of fast-growing opportunists such as Lhab-A1, Lhab-A2, Flavo-A2, Flavo-A3, betIII-A1, and bacII-A (Figure S8). PnecC was also a robust spring specialist in Římov, but negligible (< 1% maximum abundance) in Klíčava. At the clear-water phase, oligotrophic ultramicrobacteria such as LD12, Luna1-A2, acI-A1 (a subgroup of ‘*Ca*. Planktophila’), and acTH1-A1 from Alphaproteobacteria and Actinobacteria were abundant (Figure S9). LD12 was the most dominant group with up to nearly 50% of the relative proportion. In summer, cyanobacteria, including *Cyanobium*, *Microcystis*, and Aphanizomenon_NIES81, as well as unclassified Kapabacteriales, Aquir (‘*Ca.* Aquirestis’), *Pirellula*, and PnecB showed robust peaks in both reservoirs (Fig. S10). A few bacterial groups, such as PnecD and *Roseomonas* were summer specialists only in Římov. Lastly, fall/winter clusters were composed of potential decay specialists including *Methylobacter*, ‘*Ca.* Nitrotoga’, *Nitrosospira*, and *Chthoniobacter* (Figure S11).

**Fig. 6:**
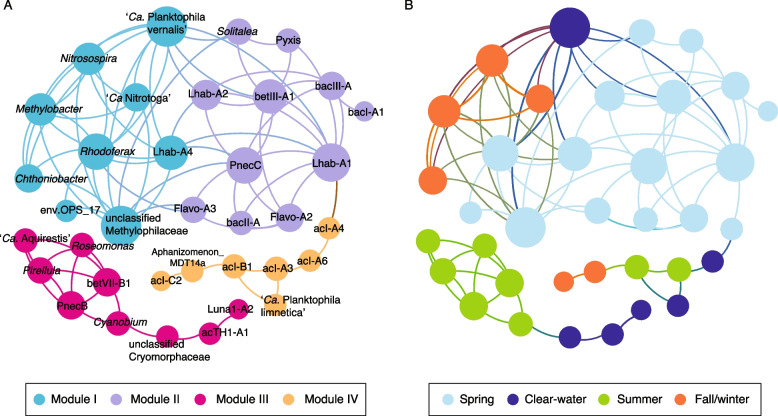
The association network of different bacterial groups. Each bacterial group is represented by a node (circle) colored according to modularity class (**A**) and seasonality (**B**). The size of each node is proportional to the number of connections (i.e., degree). The lines connecting the nodes (i.e., edges) represent strong (Spearman’s correlation coefficient > 0.5) and significant (*p* value < 0.01) correlations

An association network analysis further revealed positive correlations among the seasonally clustered bacterial groups (Fig. 6). The nodes in the network correspond to different bacterial groups, while the edges (connections between nodes) represent strong correlations (Spearman’s correlation coefficient > 0.5 and *p* value < 0.01). A total of 83 edges among 36 nodes were detected. Modularity analysis showed that the entire network could be divided into four densely connected groups (modules) (Fig. 6A). Nodes within the same module tended to belong to the same seasonal cluster (Fig. 6B). While the spring and fall/winter modules were highly interconnected to each other, the summer module was compartmentalized. Lhab-A1, PnecC, acI-A7, and unclassified Methylophilaceae played the central role in the network, showing the highest degree of associations (≥ 10). Another interesting pattern of the network was that a few actinobacterial lineages, including acI-A4, acI-A6, Phila (‘*Ca*. Planktophila limnetica’), acI-A3, acI-B1 (‘*Ca*. Nanopelagicus’), and acI-C2 showed a delayed correlation to each other across different seasons.

## Discussion

### Localized bacterial groups with indicative ecological values

The bacterial community compositions in our study involved the spatial heterogeneity between lacustrine and riverine environments, epilimnion and hypolimnion, as well as high phosphorus and sulfur-oxidizing (Klíčava) versus eutrophic (Římov) hypolimnia (Fig. 3). The dominance of ultramicrobacteria, especially LD12 and acI-B1, was a strong ecological indicative of lacustrine environments. While ultramicrobacterial populations in riverine environments have been rarely monitored, there is a consensus that the lack of grazing [15] and continuous terrestrial loading [69] do not favor their survival strategies. Our results go further in showing that riverine environments favor fast-growing bacteria, including Rhodo, Lhab-A3, PnecC, and Flavo-A3. This is in accordance with previous findings of a strong correspondence of Lhab-A3 to allochthonous dissolved organic matter (DOM) [69], PnecC to high humic contents [70], and Rhodo and Flavo-A3 to riverine conditions [37].

Water depth was often considered one of the primary factors for spatial community heterogeneities [8, 71]. The identified members of deep-water inhabitants in our study are potential key players of carbon (by *Methylobacter* and unclassified Methylophilaceae) [29, 72], nitrogen (by *Nitrosospira*) [73], or metal cycles (by Rhodo) [74]. We further observed physicochemical (Fig. 1B) and community divergence (Fig. 3) across different hypolimnia. The anoxia persisting throughout the whole year and the availability of phosphorus in the Klíčava-hypolimnion highly resembled metabolic scenarios typical for benthic habitats [75]. The occurrence of potential phosphorus-accumulating ‘*Ca.* Accumulibacter’ and many sulfur-cycling species (Fig. 3) is also in line with previous studies in anoxic conditions [76, 77]. The selectively present oligotypes affiliated with Bacteroidetes_VC2.1_Bac22 have rarely been described in pelagic habitats except in a few studies affiliating the presence of this group to extreme conditions, including hydrothermal vents at the sea bottoms [78] or a desert oasis [79]. Their distribution has been suggested in oxygen-deficient and sulfide-rich environments, and they have the potential to degrade polymers with high molecular weights and obtain energy via NO/N_2_O and polysulfide reduction [80]. In the nutrient-rich hypolimnion of the Římov reservoir, the selective occurrence of CL500-3, unclassified Isosphaeraceae, and Gemmataceae from Planctomycetota was noticeable (Fig. 3). These bacteria are well-known freshwater groups from oxygenated hypolimnion [67], and potentially mediate the decomposition of phytoplankton-derived sinking aggregates [81].

### Microdiversified bacterial populations

Previous studies demonstrated that closely related genotypes tend to play similar ecological roles and have shared niche preferences [24, 82]. However, recent evidence has also suggested that microdiversification within a bacterial group reflects an ecological strategy to survive in a wide range of environments [9, 30, 64]. In our data, two phylogenetically narrow bacterial groups, betIV-A and *Rhodoferax*, contained specialized genotypes colonizing different ecological niches (Fig. 4). Members of ‘*Ca*. Methylopumilus’ (betIV-A) were previously proposed to be psychrophilic based on their negative correlation with water temperature [29, 68]. Unexpectedly, in our study, they mainly seemed to populate warm epilimnia, and the other oligotypes affiliated with unclassified Methylophilaceae from betIV-A were dominating cold hypolimnia (Fig. 4A). The discrepancy between our and previous observations [29] could result from the resolution limitations of detection methods used (CARD-FISH versus 16S rRNA amplicon sequencing). Nevertheless, our study indicates that the presence of yet unexplored microdiversification within this group remains to be resolved. *Rhodoferax* has been well-known for its metabolic diversity and ubiquity [74, 83–85]. In our study, we show their success in environments that are strongly connected to allochthonous DOM and lack of grazing pressure (riverine sites and hypolimnia) [37], as well as their taxonomical separation into two clusters consisting of generalists and hypolimnion specialists.

Since our study was based on partial 16S rRNA sequences (V4–V5 regions), we are far away from detecting the full extent of diversities within microdiversified populations. Since many freshwater bacterial species are highly conserved in their 16S rRNA sequences, their cryptic radiation has to be uncovered by multilocus sequencing or metagenomics [86]. A few studies of comparative genomics for ‘*Ca.* Nanopelagicales’ (acI Actinobacteria) [30], *Polynucleobacter* [64], or diverse bacterial MAGs [87] successfully demonstrated that ecological diversification can overtake the ribosomal phylogenetics by horizontally acquired genes into ‘genomic islands’. Currently, one can only speculate on the speed and consequences of these evolutionary processes and their relevance for bacterial community composition and functionality conclusions.

### Generalized seasonal succession of freshwater bacteria in lacustrine environments

A temperate climate modulates consecutive annual changes in temperature and solar irradiation intensity resulting in seasonal adaptation of organisms, and their cycling in abundance and activity within a year. Such annual dynamics of freshwater bacteria have been demonstrated earlier by a few studies in lakes using k-means clustering of 16S rRNA data [24] or CARD-FISH analysis [82]. The comparison of our results with prior studies indicates that different methodologies to detect bacterial seasonality provide similar trends for those organisms showing strong seasonal preferences (e.g., Flavo-A2, Flavo-A3 for spring bloom, or PnecB and *Mycrocystis* for summer). However, it has also been reported that microbial seasonality tends to be more readily detected using soft clustering than other methods [88]. Since the most abundant bacterial groups, such as LD12, LD28, and acI lineages are highly ecologically diversified and can survive a wide range of environmental conditions, they might show slightly different [24] or weak [82] seasonal preferences in other studies. Altogether, we are convinced that this is the first study that provides both recurrent (2-year survey) and comprehensive (58 different bacterial groups) observations of how community diverges at the temporal scale.

Based on the original PEG model and our bacterial seasonality observations, we assembled an integrated model of plankton succession that connects all three major components of the microbial food web, including zooplankton, phytoplankton, and bacteria (Fig. 5 and Figure S7). In spring, the succession of fast-growing bacteria can be explained by the strong effects of increased water temperature and inorganic nutrient availability (Fig. 1C and Figure S2). The dominance of fast-growing bacteria is of short duration since lacustrine environments do not favor their survival and these opportunistic populations seem to be rapidly eliminated by selective HNF grazing, which increases the carbon flow to higher trophic levels [13, 89]. Conversely, oligotrophic ultramicrobacteria seem to be more resistant to protistan grazing and zooplankton filtration [13, 90], and adapted to low nutrient regimes [82] including the clear-water phase. In addition to well-known PnecB and PnecD [70, 91], we identified several previously overlooked heterotrophic groups, including ‘*Ca.* Aquirestis’ and *Pirellula* as the summer specialists. Previously described associations of *Pirellula* with diatom blooms [92] indicate that algae are the key prerequisite for their summer appearance. Generally, phytoplankton plays an important role in bacterial community assembly directly by the production of extracellular organic matters [93], or indirectly by the creation of attachment sites for predatory HNF [94] and the production of secondary metabolites (e.g. microcystins and lipopeptides) toxifying the grazer communities [95]. During the fall/winter periods, the succession of decay specialists coincided with a drop in temperature, chlorophyll *a*, and bacterial biomass. Their survival was considerably restricted to this season as well as hypolimnia, hinting at their psychrophilic lifestyle.

It is worth noting that we observed a pronounced zooplankton peak at the beginning of our sampling in the oligotrophic Klíčava (Fig. 5C). Since zooplankton peaks develop after some delay in response to the availability of edible phytoplankton [17], the most likely explanation for this observation is an early diatom bloom prior to our sampling period (January–February). Despite recent findings expanding our knowledge on the importance of lake winter ecology for plankton dynamics in the growing season [96, 97], winter plankton dynamics remains understudied in the current study due to technical and administrative constraints. Thus, future investigations should pay more attention to winter plankton dynamics and their implications on the PEG model.

Temporal dynamics and the co-occurrence of different freshwater bacteria were further resolved in an association network (Fig. 6). The strong correlations between 36 bacterial groups were highly supported by their seasonal relatedness. The compartmentalization of the summer module corresponded to the highest community heterogeneity in summer, which was in line with the hierarchical clustering analysis (Fig. 2). We also noticed that the delayed correlation of actinobacterial lineages in the network corroborates a previous study reporting a similar time-lagged correlation between acI-B1, acI-A3, and acI-A4 [24].

## Conclusions

Overall, the present spatiotemporal microbial survey targeting multiple recognizable zones along the longitudinal and vertical axes for two consecutive years successfully resolved the reservoir-specific microbial community dynamics. The presented spatial niche partitioning of bacterial populations has important implications for the prediction of microbial responses to different environmental conditions. The integration of bacterial seasonality into the existing PEG model represents a critical step toward a better understanding of the highly complex trophic cascading and microbial food web dynamics in freshwater ecosystems.

## Supplementary Information

**Additional file 1:** **Figure S1.** Box plots for selected physicochemical parameters measured across the sampling stations. **Figure S2.** Line plots for selected physicochemical parameters measured at the Dam regions of different reservoirs. **Figure S3.** Two-year vertical distribution of temperature in three reservoirs. **Figure S4.** Two-year vertical distribution of dissolved oxygen concentration. **Figure S5.** Rarefaction and species accumulation curves for the 310 samples estimated at oligotype-level. **Figure S6.** Hierarchical clustering of all samples (*n*=310). **Figure S7.** Annual succession patterns of planktonic organisms in reservoir ecosystems during the year 2018. **Figure S8.** Two-year temporal patterns of the individual bacterial groups from the spring cluster. **Figure S9.** Two-year temporal patterns of the individual bacterial groups from the clear-water cluster. **Figure S10.** Two-year temporal patterns of the individual bacterial groups from the summer cluster. **Figure S11.** Two-year temporal patterns of the individual bacterial groups from the fall/winter cluster.

**Additional file 2:** **Supplementary Table S1.** Indicator Species Analysis for 13 different sampling stations.

**Additional file 3:** **Supplementary Table S2.** Annual-scale dynamics of bacterial groups using soft clustering.

## Data Availability

The sequencing data from this study were deposited to the NCBI Sequence Read Archive (SRA) under BioProject PRJNA830656. R-script and the datasets necessary to run the analysis are available in Dryad repository (https://doi.org/10.5061/dryad.bg79cnpdd). All other relevant data supporting the findings of this study are available within the paper and its supplementary information files.

## Abbreviations

PEG: Plankton ecology group
DO: Dissolved oxygen
DRP: Dissolved reactive phosphorus
TP: Total phosphorus
DP: Dissolved phosphorus
DOC: Dissolved organic carbon
DN: Dissolved nitrogen
HNF: Heterotrophic nanoflagellate
MED: Minimum entropy decomposition
DOM: Dissolved organic matter

## References

[1] Ho M, Lall U, Allaire M, Pal I, Raff D, Wegner D (2017). The future role of dams in the United States of America. Water Resour Res Comment..

[2] Maavara T, Chen Q, Van Meter K, Brown LE, Zhang J, Ni J (2020). River dam impacts on biogeochemical cycling. Nat Rev Earth Environ.

[3] Carneiro FM, Bini LM (2020). Revisiting the concept of longitudinal gradients in reservoirs. Acta Limnol Bras.

[4] Thornton KW, Kennedy RH, Carroll JH, Walker WW, Gunkel RC, Ashby S (1981). Reservoir sedimentation and water quality - an heuristic model. Water Sci Technol..

[5] Yu Z, Yang J, Amalfitano S, Yu X, Liu L (2014). Effects of water stratification and mixing on microbial community structure in a subtropical deep reservoir. Sci Rep.

[6] Liu M, Zhang Y, Shi K, Zhu G, Wu Z, Liu M (2019). Thermal stratification dynamics in a large and deep subtropical reservoir revealed by high-frequency buoy data. Sci Total Environ.

[7] Lu L, Tang Q, Li H, Li Z (2022). Damming river shapes distinct patterns and processes of planktonic bacterial and microeukaryotic communities. Environ Microbiol.

[8] Paver SF, Newton RJ, Coleman ML (2020). Microbial communities of the Laurentian Great Lakes reflect connectivity and local biogeochemistry. Environ Microbiol.

[9] Okazaki Y, Fujinaga S, Salcher MM, Callieri C, Tanaka A, Kohzu A (2021). Microdiversity and phylogeographic diversification of bacterioplankton in pelagic freshwater systems revealed through long-read amplicon sequencing. Microbiome.

[10] Šimek K, Comerma M, García JC, Nedoma J, Marcé R, Armengol J (2011). The effect of river water circulation on the distribution and functioning of reservoir microbial communities as determined by a relative distance approach. Ecosystems.

[11] Pan Y, Guo S, Li Y, Yin W, Qi P, Shi J (2018). Effects of water level increase on phytoplankton assemblages in a drinking water reservoir. Water (Switzerland).

[12] Rychtecký P, Znachor P (2011). Spatial heterogeneity and seasonal succession of phytoplankton along the longitudinal gradient in a eutrophic reservoir. Hydrobiologia.

[13] Šimek K, Nedoma J, Znachor P, Kasalický V, Jezbera J, Horňák K (2014). A finely tuned symphony of factors modulates the microbial food web of a freshwater reservoir in spring. Limnol Oceanogr.

[14] Znachor P, Visocká V, Nedoma J, Rychtecký P (2013). Spatial heterogeneity of diatom silicification and growth in a eutrophic reservoir. Freshw Biol.

[15] Šimek K, Horňák K, Jezbera J, Nedoma J, Znachor P, Hejzlar J (2008). Spatio-temporal patterns of bacterioplankton production and community composition related to phytoplankton composition and protistan bacterivory in a dam reservoir. Aquat Microb Ecol.

[16] Mašín M, Jezbera J, Nedoma J, Straškrabová V, Hejzlar J, Šimek K (2003). Changes in bacterial community composition and microbial activities along the longitudinal axis of two canyon-shaped reservoirs with different inflow loading. Hydrobiologia.

[17] Sommer U, Adrian R, De Senerpont Domis L, Elser JJ, Gaedke U, Ibelings B (2012). Beyond the plankton ecology group (PEG) model: mechanisms driving plankton succession. Annu Rev Ecol Evol Syst.

[18] Romagnan JB, Legendre L, Guidi L, Jamet JL, Jamet D, Mousseau L (2015). Comprehensive model of annual plankton succession based on the whole-plankton time series approach. PLoS One.

[19] Kent AD, Yannarell AC, Rusak JA, Triplett EW, McMahon KD (2007). Synchrony in aquatic microbial community dynamics. ISME J.

[20] Zhang Y, Liu WT (2019). The application of molecular tools to study the drinking water microbiome–Current understanding and future needs. Crit Rev Environ Sci Technol..

[21] Zwart G, Crump BC, Kamst-van Agterveld MP, Hagen F, Han SK (2002). Typical freshwater bacteria: An analysis of available 16S rRNA gene sequences from plankton of lakes and rivers. Aquat Microb Ecol.

[22] Newton RJ, Jones SE, Eiler A, McMahon KD, Bertilsson S (2011). A guide to the natural history of freshwater lake bacteria. Microbiol Mol Biol Rev.

[23] Yan Q, Bi Y, Deng Y, He Z, Wu L, Van Nostrand JD (2015). Impacts of the Three Gorges Dam on microbial structure and potential function. Sci Rep.

[24] Eiler A, Heinrich F, Bertilsson S (2012). Coherent dynamics and association networks among lake bacterioplankton taxa. ISME J.

[25] Diao M, Sinnige R, Kalbitz K, Huisman J, Muyzer G (2017). Succession of bacterial communities in a seasonally stratified lake with an anoxic and sulfidic hypolimnion. Front Microbiol.

[26] Phillips AA, Speth DR, Miller LG, Wang XT, Wu F, Medeiros PM (2021). Microbial succession and dynamics in meromictic Mono Lake California. Geobiology.

[27] Jezbera J, Jezberová J, Koll U, Horňák K, Šimek K, Hahn MW (2012). Contrasting trends in distribution of four major planktonic betaproteobacterial groups along a pH gradient of epilimnia of 72 freshwater habitats. FEMS Microbiol Ecol.

[28] Salcher MM, Pernthaler J, Posch T (2011). Seasonal bloom dynamics and ecophysiology of the freshwater sister clade of SAR11 bacteria that rule the waves (LD12). ISME J.

[29] Salcher MM, Neuenschwander SM, Posch T, Pernthaler J (2015). The ecology of pelagic freshwater methylotrophs assessed by a high-resolution monitoring and isolation campaign. ISME J.

[30] Neuenschwander SM, Ghai R, Pernthaler J, Salcher MM (2018). Microdiversification in genome-streamlined ubiquitous freshwater Actinobacteria. ISME J.

[31] Flynn KJ, Stoecker DK, Mitra A, Raven JA, Glibert PM, Hansen PJ (2013). Misuse of the phytoplankton-zooplankton dichotomy: the need to assign organisms as mixotrophs within plankton functional types. J Plankton Res.

[32] Princiotta SDV, Sanders RW (2017). Heterotrophic and mixotrophic nanoflagellates in a mesotrophic lake: abundance and grazing impacts across season and depth. Limnol Oceanogr.

[33] Romano F, Symiakaki K, Pitta P (2021). Temporal variability of planktonic ciliates in a coastal oligotrophic environment: mixotrophy, size classes and vertical distribution. Front Mar Sci.

[34] International Organization for Standardization. Water Quality: Measurement of Biochemical Parameters: Spectrometric Determination of the Chlorophyll-a Concentration (ISO Standard No. 10260:1992). 1992. https://www.iso.org/standard/18300.html.

[35] Murphy J, Riley JP (1962). A modified single solution method for the determination of phosphate in natural waters. Anal Chim Acta.

[36] Kopacek J, Hejzlar J (1993). Semi-micro determination of total phosphorus in fresh waters with perchloric acid digestion. Int J Environ Anal Chem.

[37] Shabarova T, Salcher MM, Porcal P, Znachor P, Nedoma J, Grossart HP (2021). Recovery of freshwater microbial communities after extreme rain events is mediated by cyclic succession. Nat Microbiol.

[38] Sherr EB, Sherr BF, Kemp PF, Sherr BF, Sherr EB, Cole JJ (1993). Preservation and storage of samples for enumeration of heterotrophic protists. Handbook of methods in aquatic microbial ecology.

[39] Porter KG, Feig YS (1980). The use of DAPI for identifying and counting aquatic microflora. Limnol Oceanogr.

[40] Lund JWG, Kipling C, Le Cren ED (1958). The inverted microscope method of estimating algal numbers and the statistical basis of estimations by counting. Hydrobiologia.

[41] Hillebrand H, Dürselen CD, Kirschtel D, Pollingher U, Zohary T (1999). Biovolume calculation for pelagic and benthic microalgae. J Phycol.

[42] McCauley E, Edmonson WT (1984). The estimation of the abundance and biomass of zooplankton insamples. A manual on methods for the assessment of secondary productivity in freshWaters.

[43] Minas K, Mcewan NR, Newbold CJ, Scott KP (2011). Optimization of a high-throughput CTAB-based protocol for the extraction of qPCR-grade DNA from rumen fluid, plant and bacterial pure cultures. FEMS Microbiol Lett.

[44] Walters W, Hyde ER, Berg-Lyons D, Ackermann G, Humphrey G, Parada A (2016). Improved bacterial 16S rRNA gene (V4 and V4-5) and fungal internal transcribed spacer marker gene primers for microbial community surveys. mSystems.

[45] Parada AE, Needham DM, Fuhrman JA (2016). Every base matters: assessing small subunit rRNA primers for marine microbiomes with mock communities, time series and global field samples. Environ Microbiol.

[46] Callahan BJ, McMurdie PJ, Rosen MJ, Han AW, Johnson AJA, Holmes SP (2016). DADA2: High-resolution sample inference from Illumina amplicon data. Nat Methods.

[47] Eren AM, Morrison HG, Lescault PJ, Reveillaud J, Vineis JH, Sogin ML (2015). Minimum entropy decomposition: unsupervised oligotyping for sensitive partitioning of high-throughput marker gene sequences. ISME J.

[48] Rohwer RR, Hamilton JJ, Newton RJ, McMahon KD. TaxAss: Leveraging a custom freshwater database achieves fine-scale taxonomic resolution. mSphere. 2018;3(5):e00327–18. 10.1128/mSphere.00327-18 PMC6126143 30185512

[49] Quast C, Pruesse E, Yilmaz P, Gerken J, Schweer T, Yarza P (2013). The SILVA ribosomal RNA gene database project: Improved data processing and web-based tools. Nucleic Acids Res.

[50] Yilmaz P, Parfrey LW, Yarza P, Gerken J, Pruesse E, Quast C (2014). The SILVA and “all-species Living Tree Project (LTP)” taxonomic frameworks. Nucleic Acids Res.

[51] McMurdie PJ, Holmes S. Phyloseq: an R package for reproducible interactive analysis and graphics of microbiome census data. PLoS One. 2013;8(4):e61217. 10.1371/journal.pone.0061217 PMC3632530 23630581

[52] Kandlikar GS, Gold ZJ, Cowen MC, Meyer RS, Freise AC, Kraft NJB (2018). ranacapa: An R package and Shiny web app to explore environmental DNA data with exploratory statistics and interactive visualizations. F1000Res.

[53] Dixon P (2003). Computer program review VEGAN, a package of R functions for community ecology. J Veg Sci.

[54] R Core Team (2020). R: A language and environment for statistical computing.

[55] De Cáceres M, Legendre P, Wiser SK, Brotons L (2012). Using species combinations in indicator value analyses. Methods Ecol Evol.

[56] Stamatakis A, Ludwig T, Meier H (2005). RAxML-II: a program for sequential, parallel and distributed inference of large phylogenetic trees. Concurr Comput Pract Exp.

[57] Katoh K, Standley DM (2013). MAFFT multiple sequence alignment software version 7: Improvements in performance and usability. Mol Biol Evol.

[58] Kumar L, Futschik ME (2007). Mfuzz: a software package for soft clustering of microarray data. Bioinformation.

[59] Hadley W (2016). ggplot2: elegant graphics for data analysis.

[60] Hu A, Ju F, Hou L, Li J, Yang X, Wang H (2017). Strong impact of anthropogenic contamination on the co-occurrence patterns of a riverine microbial community. Environ Microbiol.

[61] Bastian M, Heymann S, Jacomy M (2009). Gephi: an open source software for exploring and manipulating networks. ICWSM..

[62] Rohwer RR, Hamilton JJ, Newton RJ, McMahon KD (2018). TaxAss: leveraging a custom freshwater database achieves fine-scale taxonomic resolution. mSphere.

[63] Henson MW, Lanclos VC, Faircloth BC, Thrash JC (2018). Cultivation and genomics of the first freshwater SAR11 (LD12) isolate. ISME J.

[64] Hoetzinger M, Schmidt J, Jezberová J, Koll U, Hahn MW. Microdiversification of a pelagic Polynucleobacter species is mainly driven by acquisition of genomic islands from a partially interspecific gene pool. Appl Environ Microbiol. 2017;83(3):e02266-16. 10.1128/AEM.02266-16 PMC5244307 27836842

[65] Kasalický V, Jezbera J, Hahn MW, Šimek K. The diversity of the Limnohabitans genus, an important group of freshwater bacterioplankton, by characterization of 35 isolated strains. PLoS One. 2013;8(3):e58209. 10.1371/journal.pone.0058209 PMC3591437 23505469

[66] Flowers JJ, He S, Malfatti S, del Rio TG, Tringe SG, Hugenholtz P (2013). Comparative genomics of two ’Candidatus Accumulibacter’clades performing biological phosphorus removal. ISME J.

[67] Okazaki Y, Fujinaga S, Tanaka A, Kohzu A, Oyagi H, Nakano SI (2017). Ubiquity and quantitative significance of bacterioplankton lineages inhabiting the oxygenated hypolimnion of deep freshwater lakes. ISME J.

[68] Salcher MM, Schaefle D, Kaspar M, Neuenschwander SM, Ghai R (2019). Evolution in action: habitat transition from sediment to the pelagial leads to genome streamlining in Methylophilaceae. ISME J.

[69] Shabarova T, Kasalický V, Šimek K, Nedoma J, Znachor P, Posch T (2017). Distribution and ecological preferences of the freshwater lineage LimA (genus Limnohabitans) revealed by a new double hybridization approach. Environ Microbiol.

[70] Watanabe K, Komatsu N, Kitamura T, Ishii Y, Park HD, Miyata R (2012). Ecological niche separation in the Polynucleobacter subclusters linked to quality of dissolved organic matter: a demonstration using a high sensitivity cultivation-based approach. Environ Microbiol.

[71] Linz A, Shade A, Owens S, Jack G (2017). Bacterial community composition and dynamics spanning five years in freshwater Bog lakes.

[72] van Grinsven S, Sinninghe Damsté JS, Harrison J, Polerecky L, Villanueva L (2021). Nitrate promotes the transfer of methane-derived carbon from the methanotroph Methylobacter sp. to the methylotroph Methylotenera sp. in eutrophic lake water. Limnol Oceanogr.

[73] Urakawa H, Garcia JC, Nielsen JL, Le VQ, Kozlowski JA, Stein LY (2015). Nitrosospira lacus sp. nov., a psychrotolerant, ammonia-oxidizing bacterium from sandy lake sediment. Int J Syst Evol Microbiol.

[74] Risso C, Sun J, Zhuang K, Mahadevan R, DeBoy R, Ismail W (2009). Genome-scale comparison and constraint-based metabolic reconstruction of the facultative anaerobic Fe(III)-reducer Rhodoferax ferrireducens. BMC Genomics.

[75] Tammeorg O, Nürnberg G, Niemistö J, Haldna M, Horppila J (2020). Internal phosphorus loading due to sediment anoxia in shallow areas: implications for lake aeration treatments. Aquat Sci.

[76] Diao M, Huisman J, Muyzer G (2018). Spatio-temporal dynamics of sulfur bacteria during oxic-anoxic regime shifts in a seasonally stratified lake. FEMS Microbiol Ecol.

[77] Watson SJ, Needoba JA, Peterson TD (2019). Widespread detection of Candidatus Accumulibacter phosphatis, a polyphosphate-accumulating organism, in sediments of the Columbia River estuary. Environ Microbiol.

[78] Lloyd KG, Steen AD, Ladau J, Yin J, Crosby L. Phylogenetically novel uncultured microbial cells dominate earth microbiomes. mSystems. 2018;3(5):e00055-18. 10.1128/mSystems.00055-18 PMC6156271 30273414

[79] Lee ZMP, Poret-Peterson AT, Siefert JL, Kaul D, Moustafa A, Allen AE (2017). Nutrient stoichiometry shapes microbial community structure in an evaporitic shallow pond. Front Microbiol.

[80] Leng H, Zhao W, Xiao X (2022). Cultivation and metabolic insights of an uncultured clade, Bacteroidetes VC2.1 Bac22 (Candidatus Sulfidibacteriales ord. nov.), from deep-sea hydrothermal vents. Environ Microbiol.

[81] Andrei AŞ, Salcher MM, Mehrshad M, Rychtecký P, Znachor P, Ghai R (2019). Niche-directed evolution modulates genome architecture in freshwater Planctomycetes. ISME J.

[82] Salcher MM (2014). Same same but different: ecological niche partitioning of planktonic freshwater prokaryotes. J Limnol.

[83] Farh MEA, Kim YJ, Singh P, Jung SY, Kang JP, Yang DC (2017). Rhodoferax koreense sp. nov, an obligately aerobic bacterium within the family Comamonadaceae, and emended description of the genus Rhodoferax. J Microbiol..

[84] Hiraishi A, Hoshino Y, Satoh T (1991). Rhodoferax fermentans gen. nov., sp. nov., a phototrophic purple nonsulfur bacterium previously referred to as the “Rhodocyclus gelatinosus-like” group. Arch Microbiol.

[85] Madigan MT, Jung DO, Woese CR, Achenbach LA (2000). Rhodoferax antarcticus sp. nov., a moderately psychrophilic purple nonsulfur bacterium isolated from an Antarctic microbial mat. Arch Microbiol.

[86] Jaspers E, Overmann J (2004). Ecological significance of microdiversity: identical 16S rRNA gene sequences can be found in bacteria with highly divergent genomes and ecophysiologies. Appl Environ Microbiol.

[87] Okazaki Y, Nakano S, Toyoda A, Tamaki H (2022). Long-read-resolved, ecosystem-wide exploration of nucleotide and structural microdiversity of lake bacterioplankton genomes. mSystems.

[88] Ward CS, Yung CM, Davis KM, Blinebry SK, Williams TC, Johnson ZI (2017). Annual community patterns are driven by seasonal switching between closely related marine bacteria. ISME J.

[89] Jezbera J, Horňák K, Šimek K (2006). Prey selectivity of bacterivorous protists in different size fractions of reservoir water amended with nutrients. Environ Microbiol.

[90] Tarao M, Jezbera J, Hahn MW (2009). Involvement of cell surface structures in size-independent grazing resistance of freshwater Actinobacteria. Appl Environ Microbiol.

[91] Wu QL, Hahn MW (2006). High predictability of the seasonal dynamics of a species-like Polynucleobacter population in a freshwater lake. Environ Microbiol.

[92] Morris RM, Longnecker K, Giovannoni SJ (2006). Pirellula and OM43 are among the dominant lineages identified in an Oregon coast diatom bloom. Environ Microbiol.

[93] Šimek K, Kasalický V, Zapomělová E, Horňák K (2011). Alga-derived substrates select for distinct betaproteobacterial lineages and contribute to niche separation in Limnohabitans strains. Appl Environ Microbiol.

[94] Šimek K, Jezbera J, Horňák K, Vrba J, Sed’a J (2004). Role of diatom-attached choanoflagellates of the genus Salpingoeca as pelagic bacterivores. Aquat Microb Ecol..

[95] Wiegand C, Pflugmacher S (2005). Ecotoxicological effects of selected cyanobacterial secondary metabolites a short review. Toxicol Appl Pharmacol.

[96] Kong X, Seewald M, Dadi T, Friese K, Mi C, Boehrer B (2021). Unravelling winter diatom blooms in temperate lakes using high frequency data and ecological modeling. Water Res.

[97] Hampton SE, Galloway AWE, Powers SM, Ozersky T, Woo KH, Batt RD (2017). Ecology under lake ice. Ecol Lett.

