# Supplementary material for: In the right place, at the right time: the integration of bacteria into the Plankton Ecology Group model
Source: Microbiome. 2023 May 20;11:112. doi: 10.1186/s40168-023-01522-0 (PMC10199524; doi:10.1186/s40168-023-01522-0)
Supplement: Supplementary file 2 — Additional file 1: Figure S1. Box plots for selected physicochemical parameters measured across the sampling stations. Figure S2. Line plots for selected physicochemical parameters measured at the Dam regions of different reservoirs. Figure S3. Two-year vertical distribution of temperature in three reservoirs. Figure S4. Two-year vertical distribution of dissolved oxygen concentration. Figure S5. Rarefaction and species accumulation curves for the 310 samples estimated at oligotype-level. Figure S6. Hierarchical clustering of all samples (n=310). Figure S7. Annual succession patterns of planktonic organisms in reservoir ecosystems during the year 2018. Figure S8. Two-year temporal patterns of the individual bacterial groups from the spring cluster. Figure S9. Two-year temporal patterns of the individual bacterial groups from the clear-water cluster. Figure S10. Two-year temporal patterns of the individual bacterial groups from the summer cluster. Figure S11. Two-year temporal patterns of the individual bacterial groups from the fall/winter cluster. [file 40168_2023_1522_MOESM1_ESM.pdf]

In the right place, at the right time: the integration of bacteria into the Plankton Ecology Group model

Hongjae Park[1], Tanja Shabarova[1], Michaela M. Salcher[1], Lenka Kosová[1], Pavel Rychtecký[1], Indranil Mukherjee[1], Karel Šimek[1,2], Petr Porcal[1,2], Jaromír Seďa[1], Petr Znachor[1,2], Vojtěch Kasalický[1]

[1]*Institute of Hydrobiology, Biology Centre of the Czech Academy of Sciences, České Budějovice, Czech Republic.* [2]*Faculty of Science, University of South Bohemia, České Budějovice, Czech Republic.*

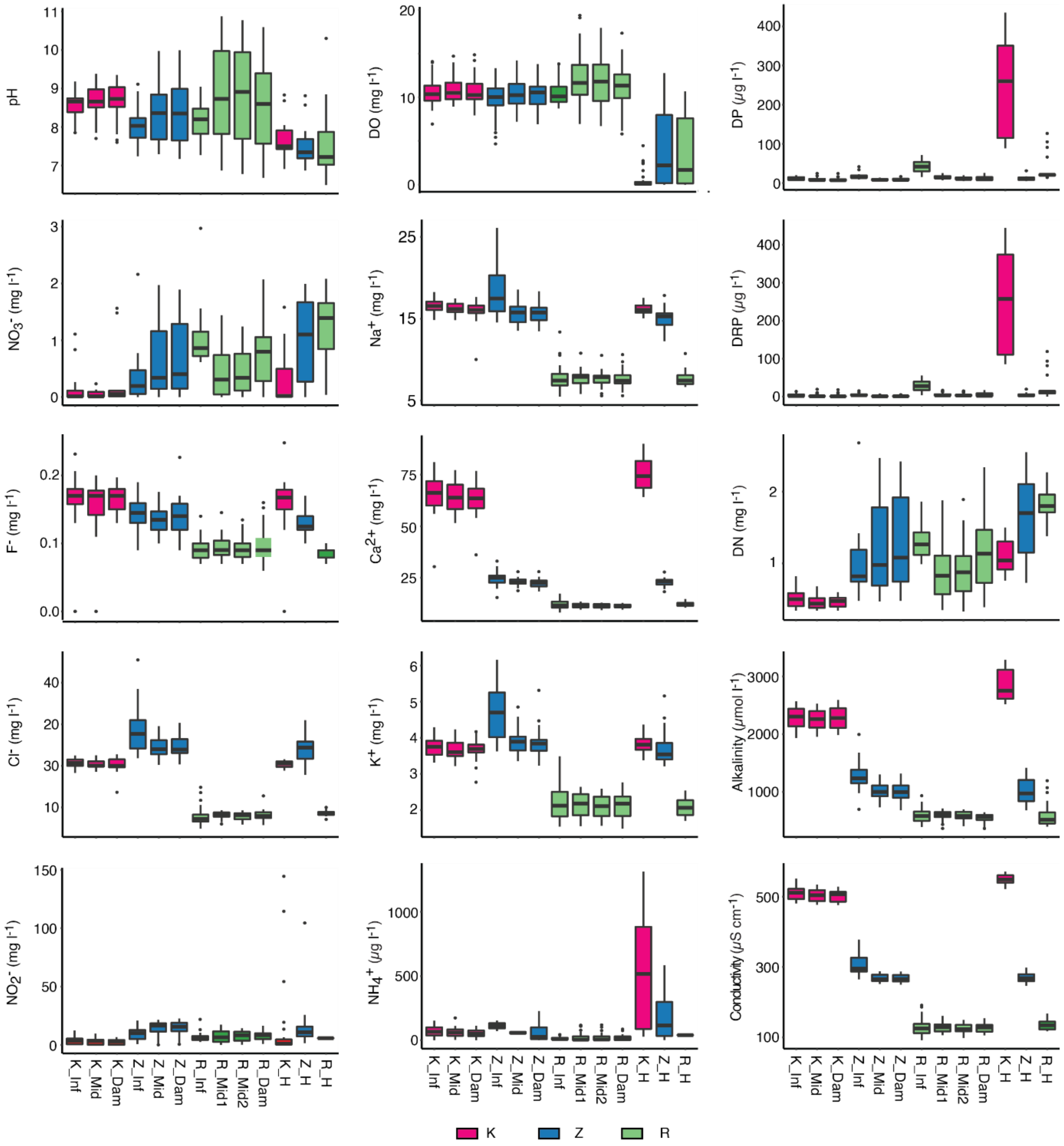

Figure S1. Box plots for selected physicochemical parameters measured across the sampling stations. Each box plot includes all the samples collected during the sampling period. The lower and upper edges of the boxplots correspond to the first and third quartiles, the whiskers extend to the largest or smallest value at 1.5 times the interquartile, and the black bars across the box represent median values. Dots that exist beyond one of the whiskers represent outliers. K: Klíčava, Z: Žlutice, R: Římov, Inf: Inflow, Mid: Middle, H: Hypolimnion

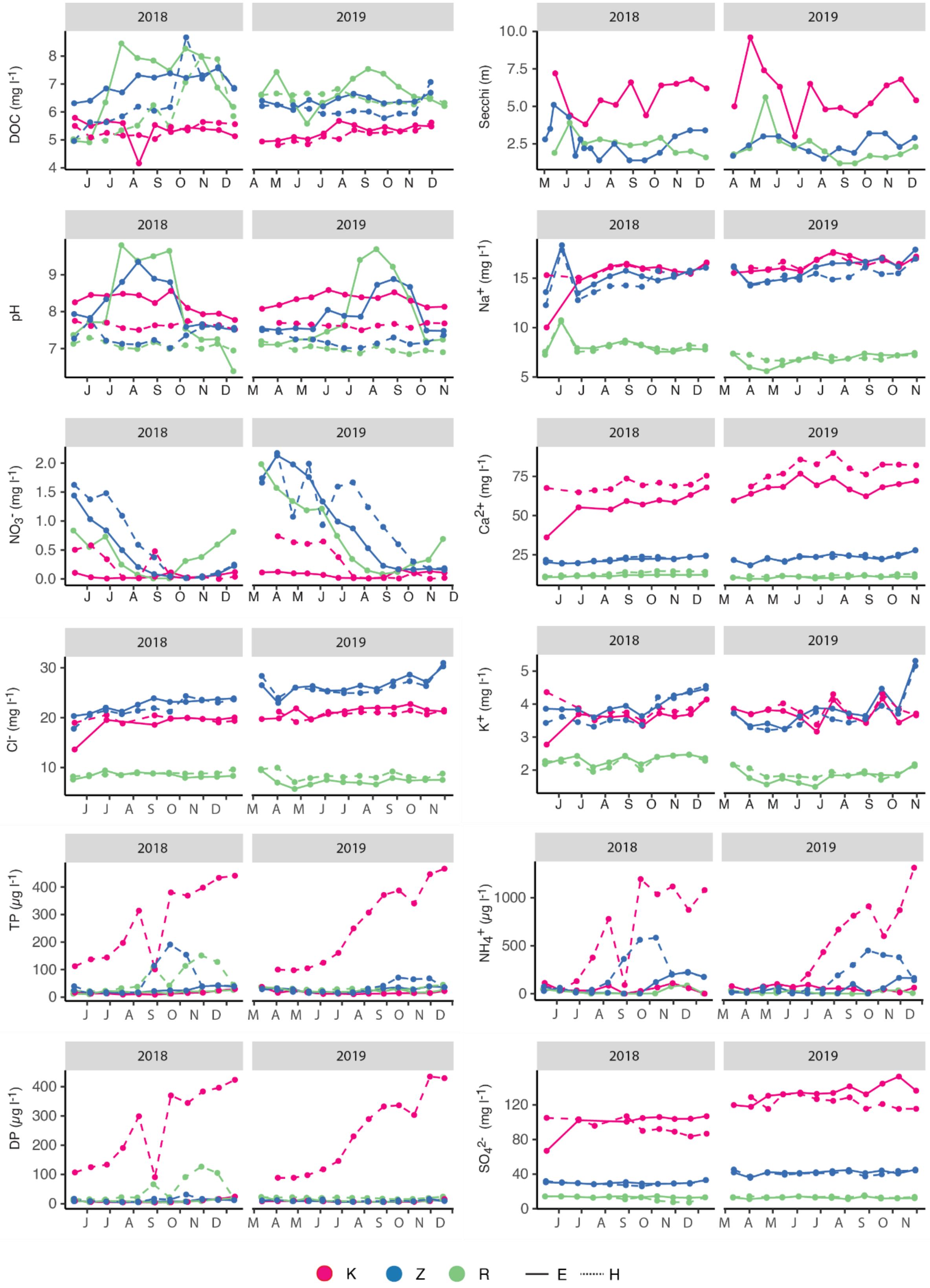

Figure S2. Line plots for selected physicochemical parameters measured at the Dam regions of different reservoirs. K: Klíčava, Z: Žlutice, R: Římov, E: Epilimnion, H: Hypolimnion

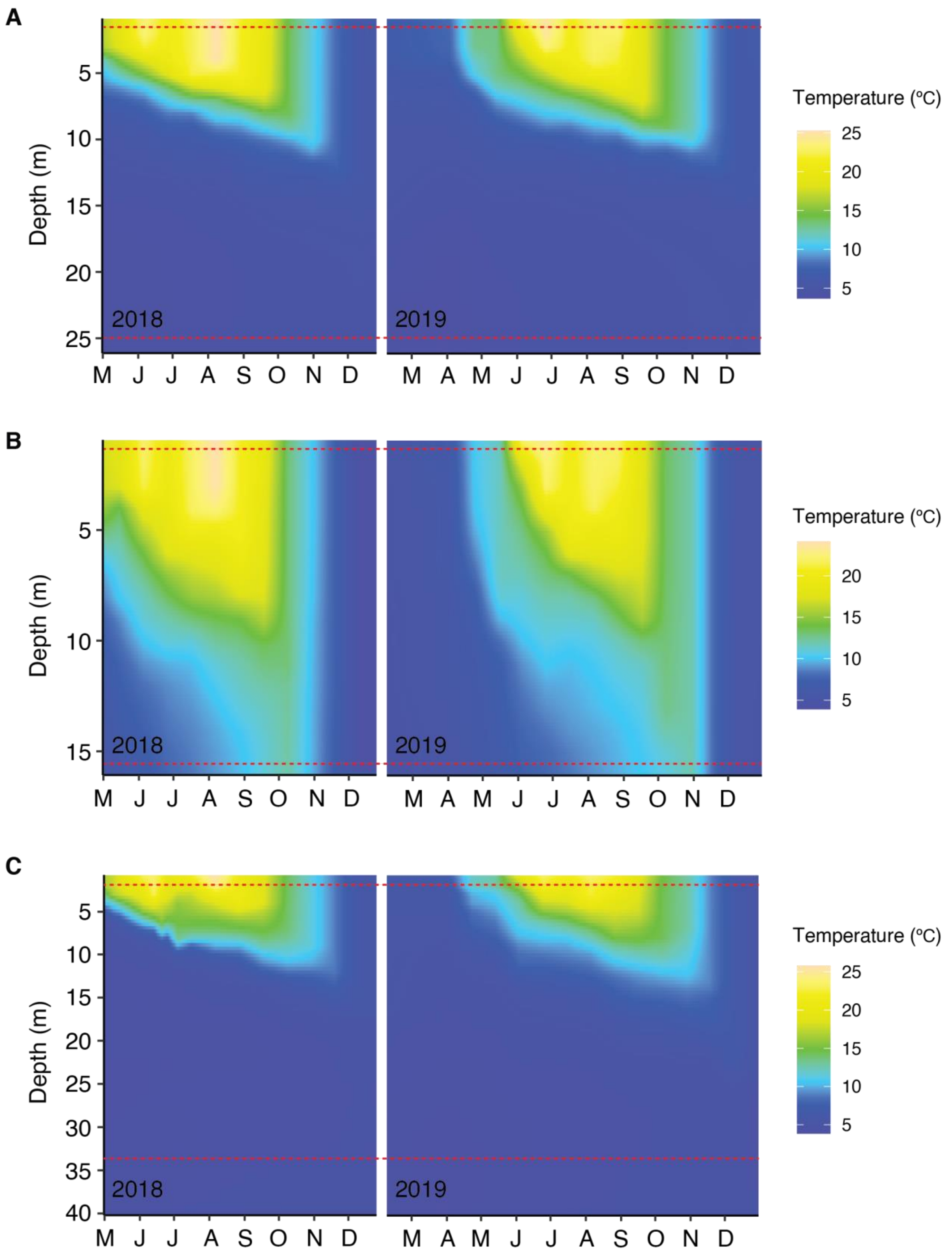

Figure S3. Two-year vertical distribution of temperature in (A) Klíčava, (B) Žlutice, and (C) Římov. Red dotted lines indicate sampling depths from different reservoirs.

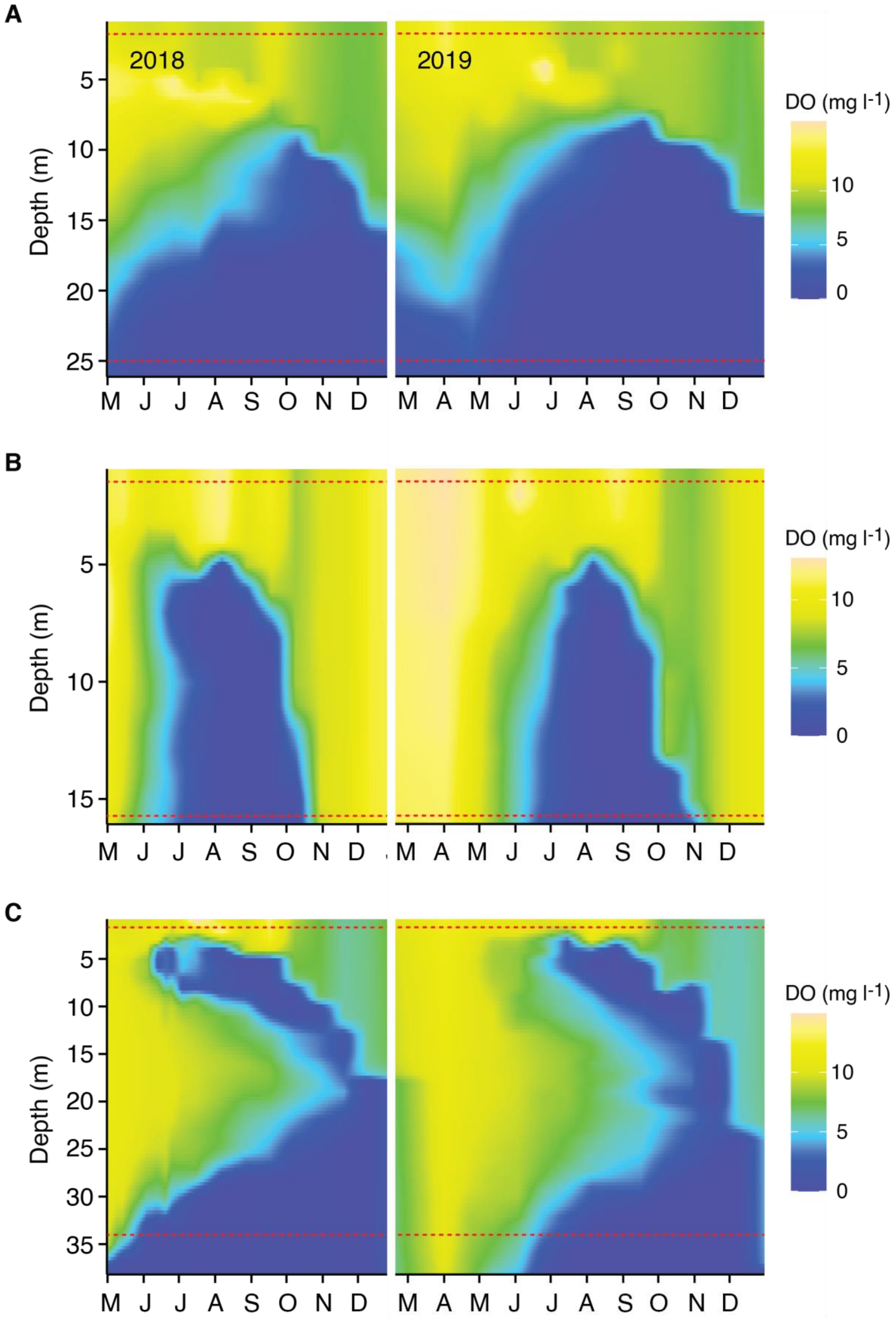

Figure S4. Two-year vertical distribution of dissolved oxygen concentration in (A) Klíčava, (B) Žlutice, and (C) Římov. Red dotted lines indicate sampling depths from different reservoirs.

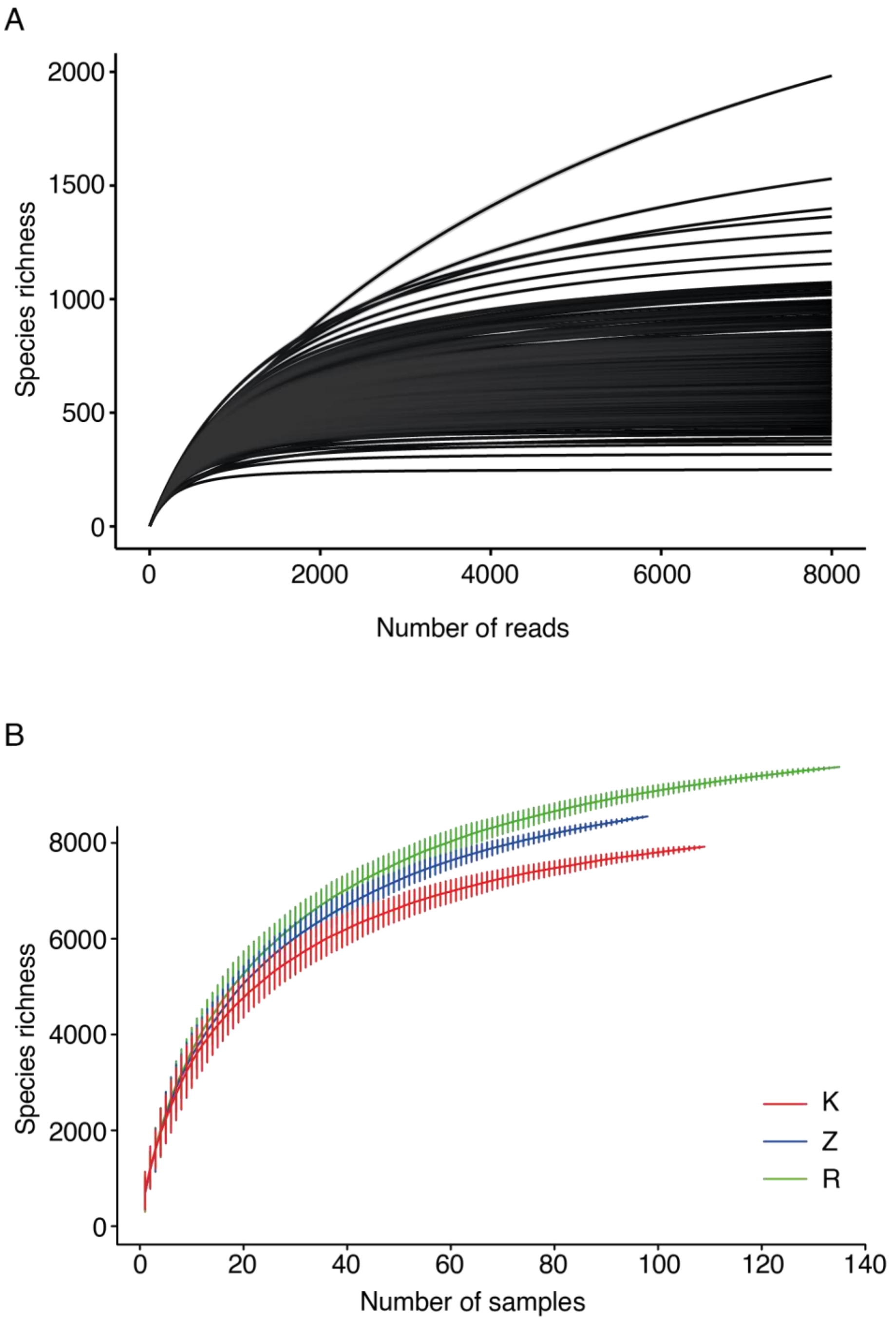

Figure S5. Rarefaction (A) and species accumulation curves (B) for the 310 samples estimated at oligotype-level. Solid lines for the species accumulation curves represent observed species richness with corresponding 95 % CI intervals (color-shaded area).

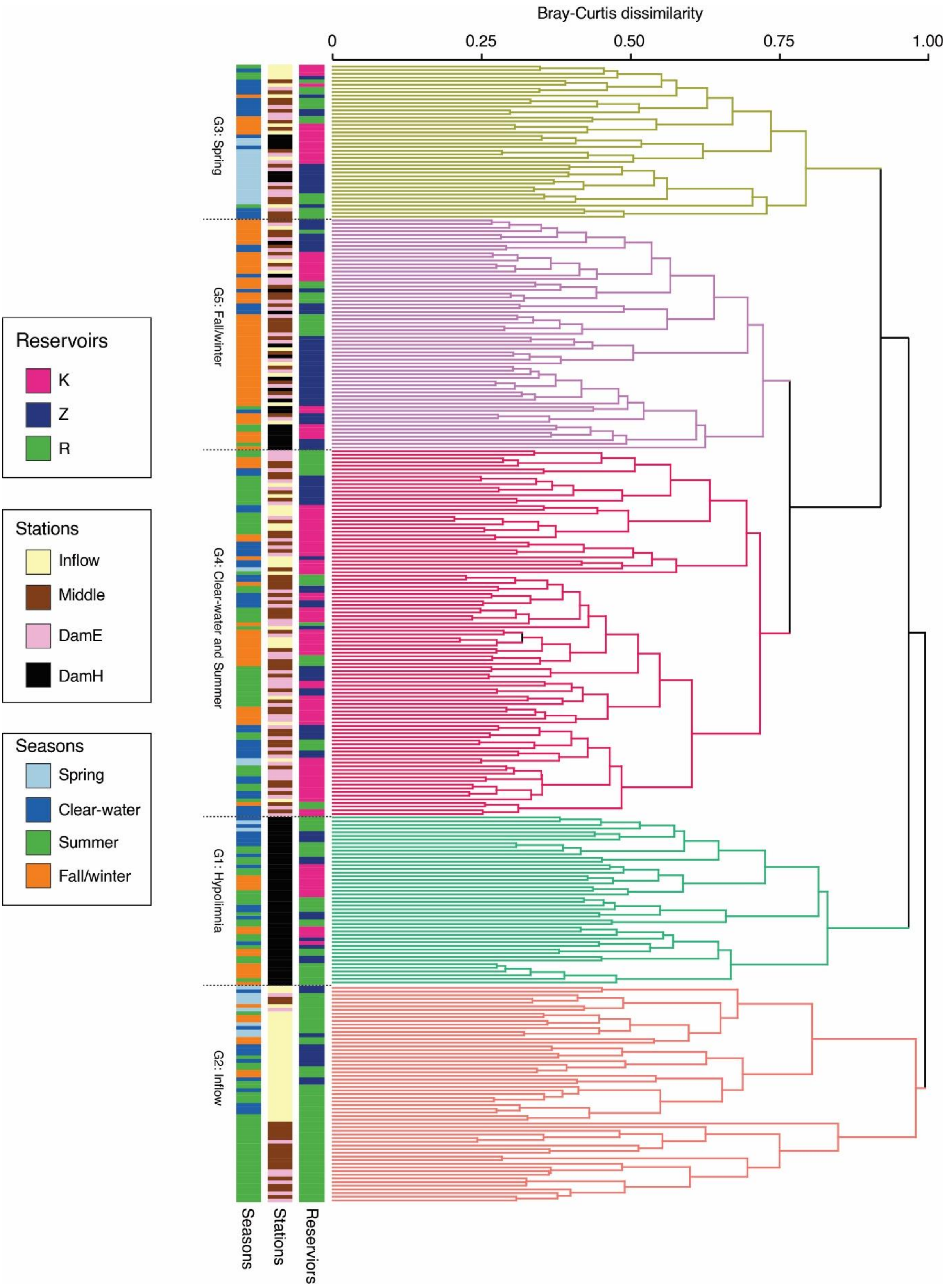

Figure S6. Hierarchical clustering of all samples (n=310). The dendrogram shows the clustering of all samples from three reservoirs (K: Klíčava, Z: Žlutice, R: Římov) covering two water depths (E: Epilimnion, H:Hypolimnion), 13 sampling stations, and four successional seasonal stages (spring, clear-water, summer, and fall/winter) over two years.

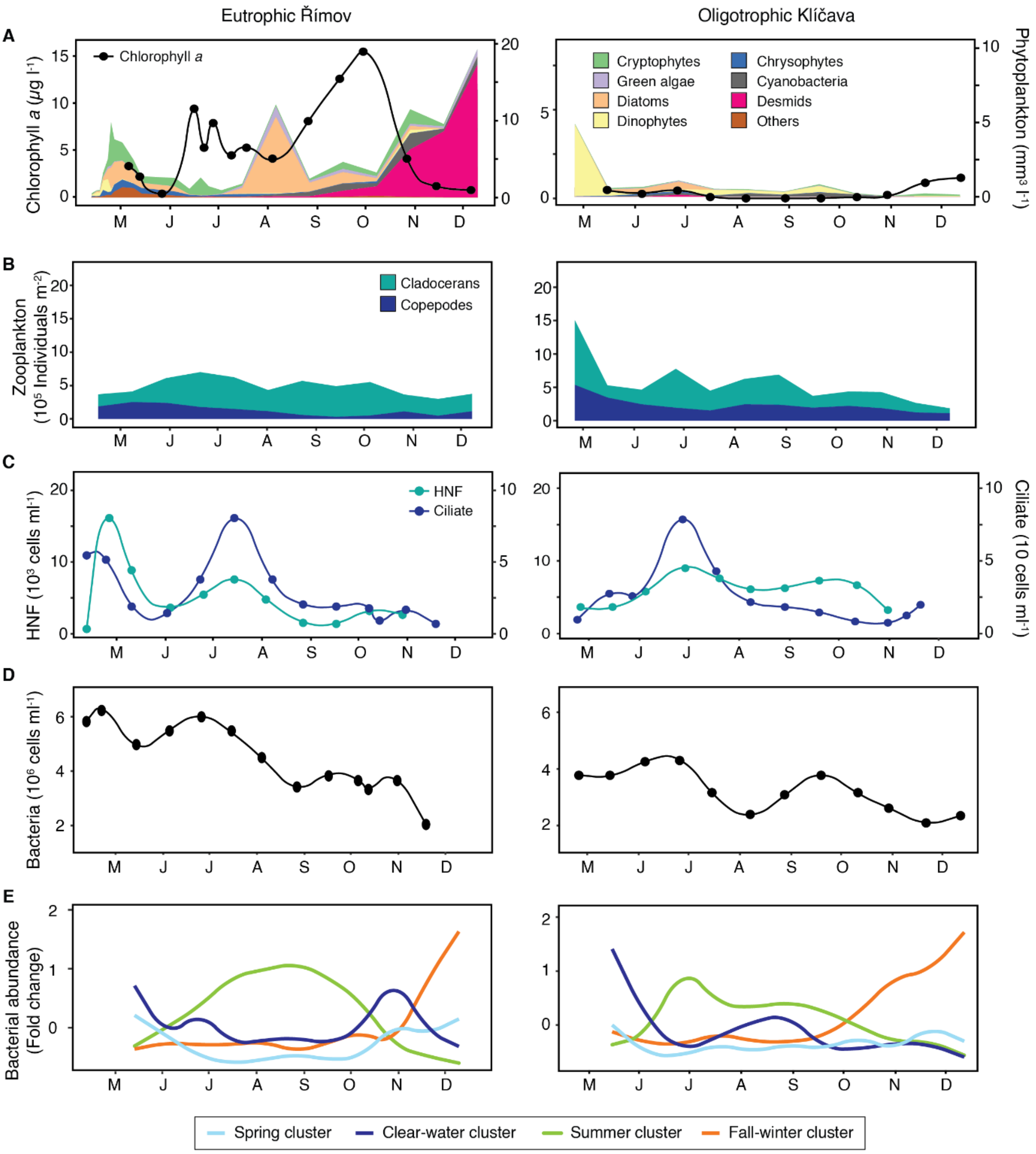

Figure S7. Annual succession patterns of planktonic organisms in reservoir ecosystems during the year 2018. (A) chlorophyll *a* concentration and phytoplankton biomass, (B) seasonality of zooplanktons, (C) seasonality of HNF and ciliates, (D) total bacterial cell counts, (E) the average abundance changes of four seasonal clusters based on a soft clustering analysis.

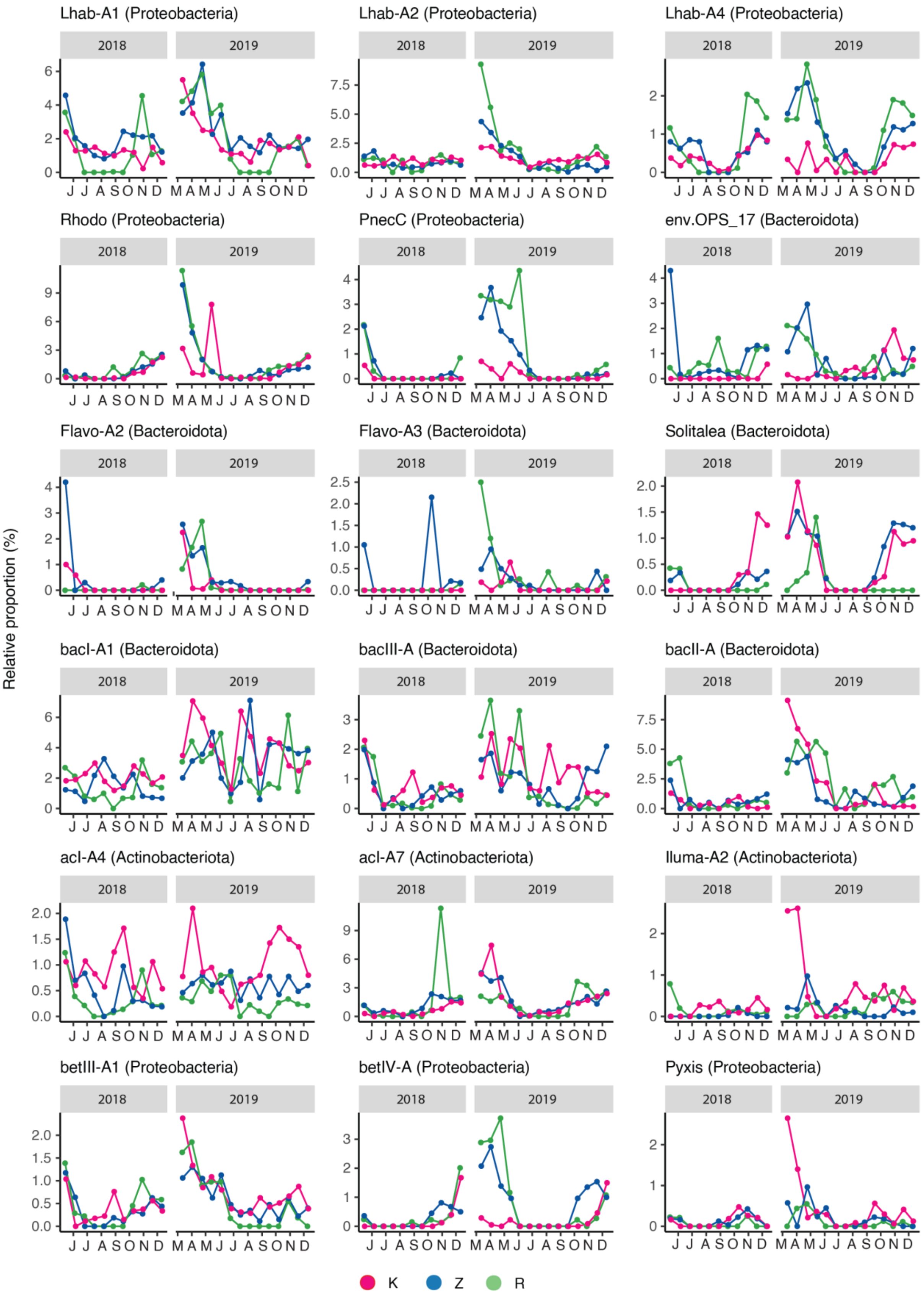

Figure S8. Two-year temporal patterns of the individual bacterial groups from the spring cluster. K: Klíčava, Z: Žlutice, R: Římov

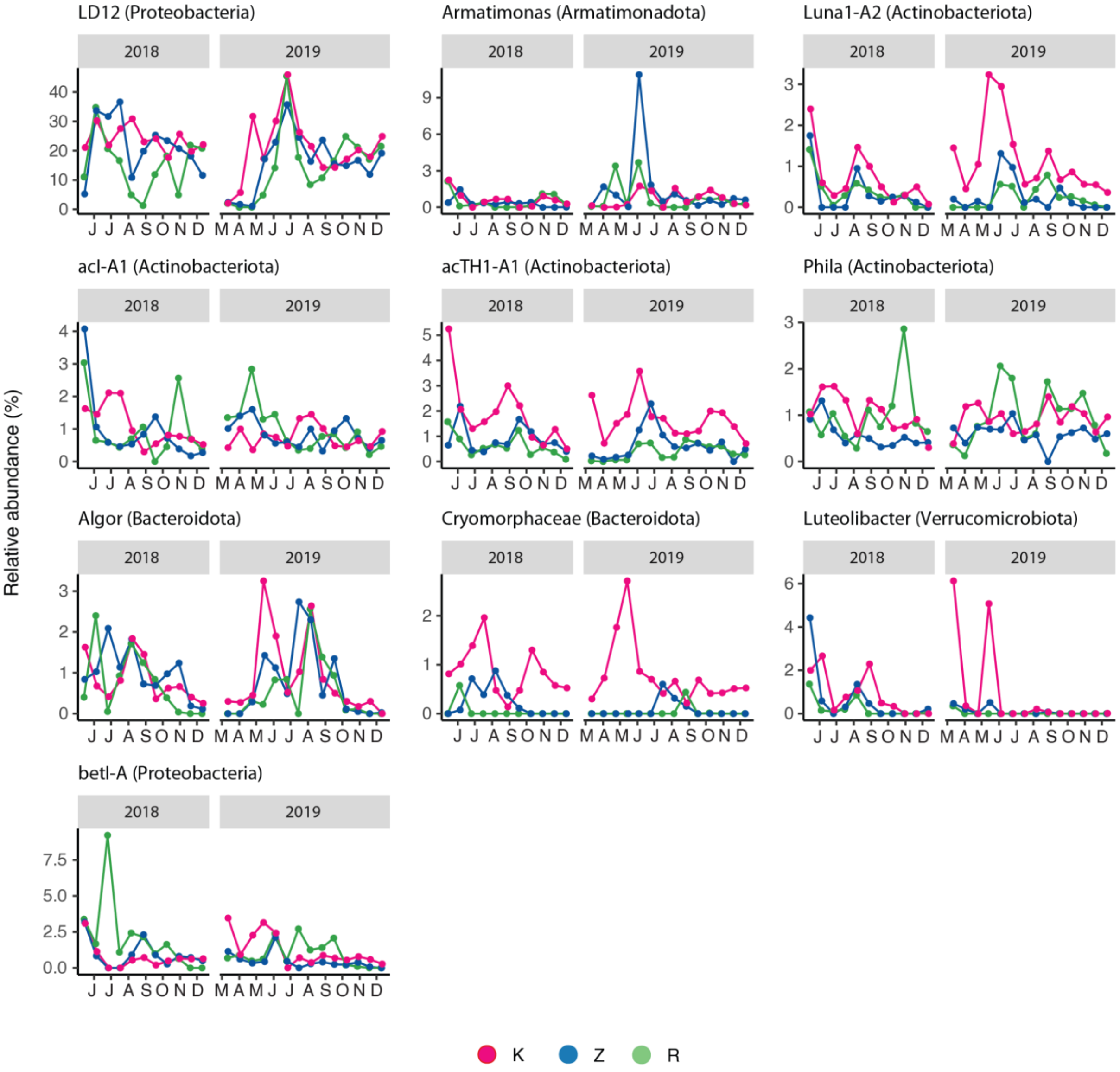

Figure S9. Two-year temporal patterns of the individual bacterial groups from the clear-water cluster. K: Klíčava, Z: Žlutice, R: Římov

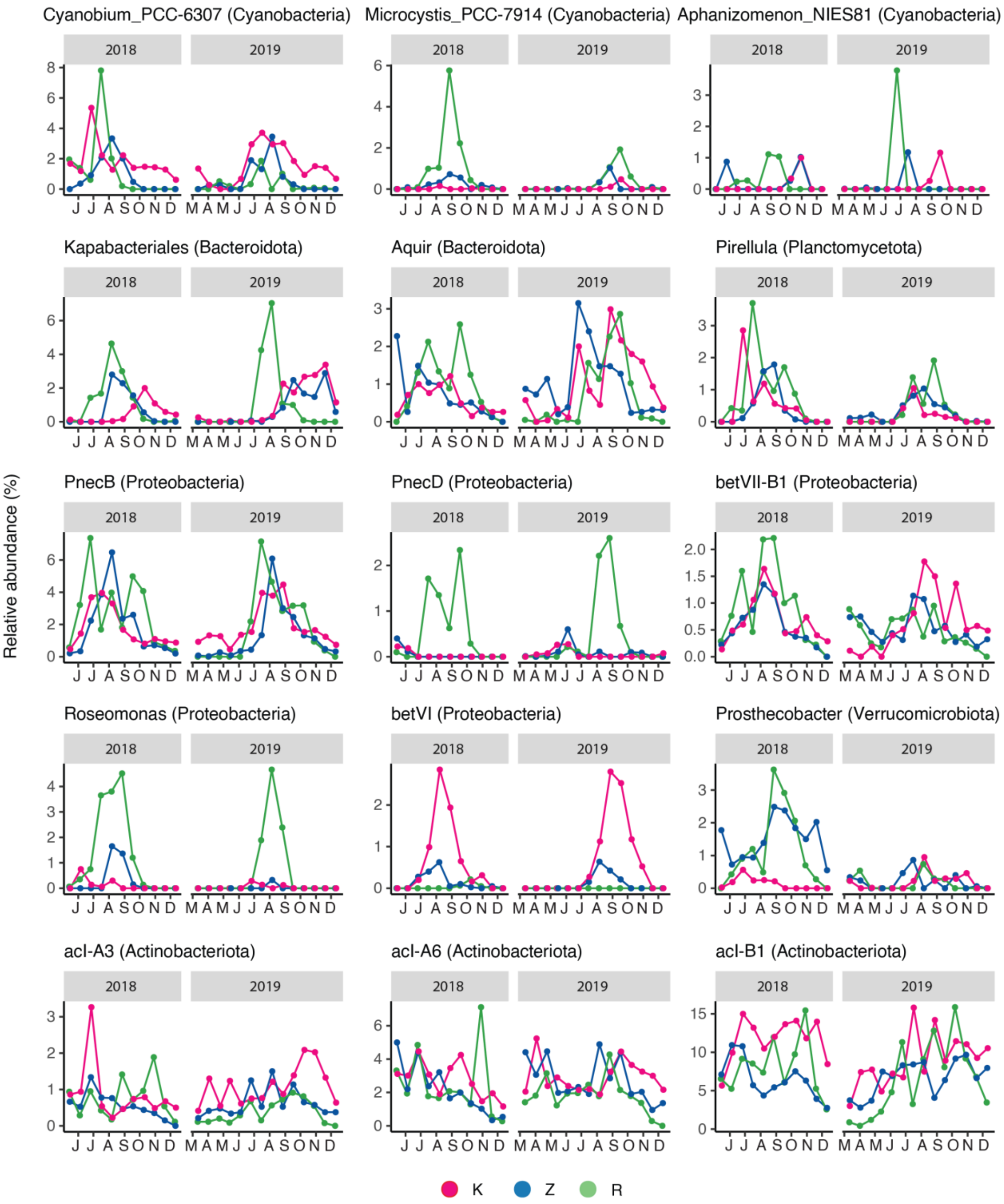

Figure S10. Two-year temporal patterns of the individual bacterial groups from the summer cluster. K: Klíčava, Z: Žlutice, R: Římov

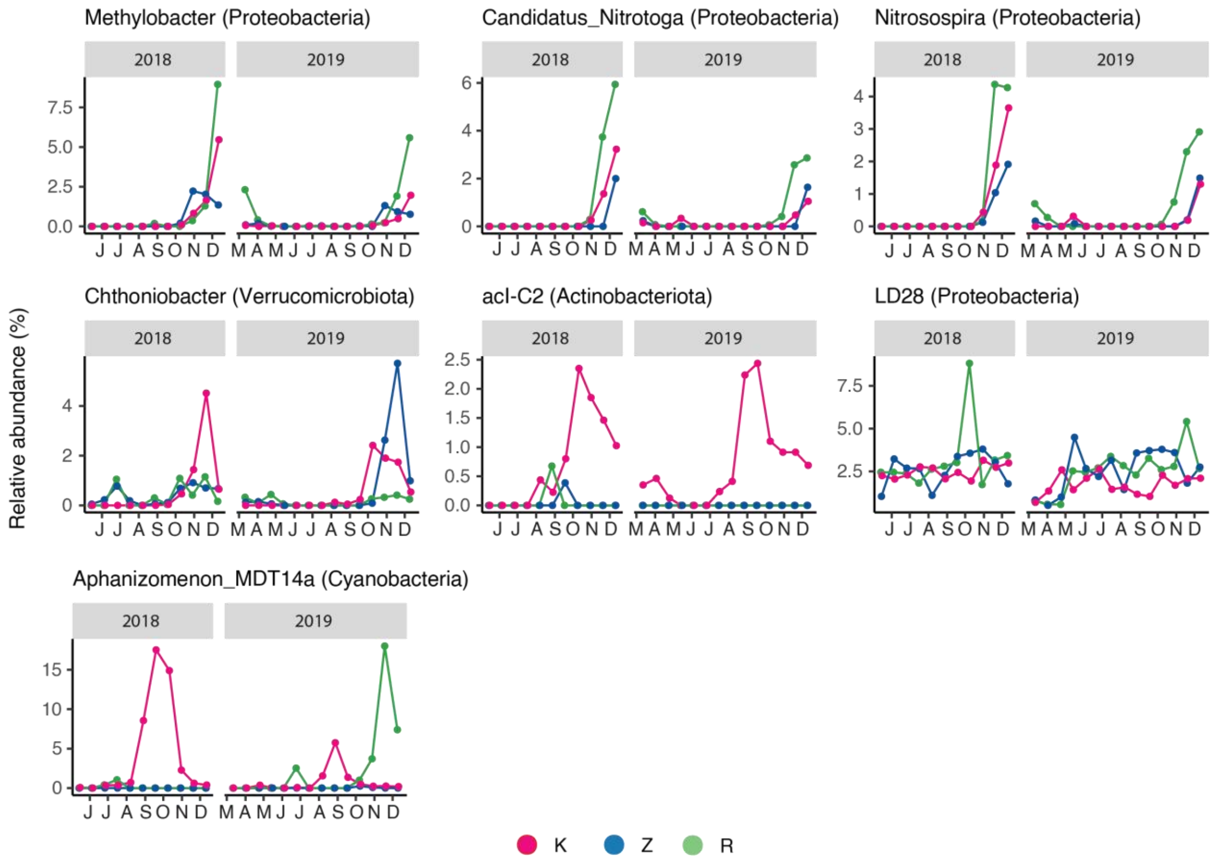

Figure S11. Two-year temporal patterns of the individual bacterial groups from the fall/winter cluster. K: Klíčava, Z: Žlutice, R: Římov
